# Rictor/mTORC2 signaling pathway protects endogenous neural stem cells to promote recovery after spinal cord injury

**DOI:** 10.4103/NRR.NRR-D-25-00544

**Published:** 2025-12-30

**Authors:** Kuileung Tong, Shiming Li, Guoliang Chen, Dacheng He, Chengkai Lin, Yuhang Li, Ningning Chen

**Affiliations:** 1 Guangdong Provincial Biomedical Innovation Platform of Regeneration and Repair of Spinal Cord and Nerve Injury, Department of Orthopedic Surgery, Shenzhen Key Laboratory of Bone Tissue Repair and Translational Research, The Seventh Affiliated Hospital, Sun Yat-sen University, Shenzhen, Guangdong Province, China; 2 Department of Orthopedics, Third Affiliated Hospital of Sun Yat-sen University, Guangzhou, Guangdong Province, China; 3 Department of Orthopedics Surgery, The Affiliated Suzhou Hospital of Nanjing Medical University, Nanjing, Jiangsu Province, China; 4 Department of Orthopedic Surgery, The First Affiliated Hospital, Jinan University, Guangzhou, Guangdong Province, China

**Keywords:** endogenous neural stem cell, glial scar, inflammation, intracellular Ca^2+^, lysosome, mTORC2, necroptosis, Rictor, RIPK1, spinal cord injury

## Abstract

Although endogenous neural stem cells represent a promising target for noninvasive spinal cord injury repair, inflammatory lesion environments frequently trigger their death. Our prior work identified necroptosis as a key death pathway for endogenous neural stem cells migrating to spinal cord injury lesions. Rapamycin-insensitive companion of mTOR (Rictor; a core component of the mechanistic target of rapamycin complex 2 [mTORC2] complex) regulates neural stem cell self-renewal and differentiation, and our preliminary data implicate it in spinal cord injury repair; however, its role in promoting endogenous neural stem cell survival post-spinal cord injury remains unclear. Here, we generated conditional endogenous neural stem cell-specific Rictor knockout mice using the Cre-loxP system. Although the endogenous neural stem cell-specific Rictor knockout mice displayed normal baseline spinal cord morphology and function, they exhibited impaired functional recovery after spinal cord injury compared with wild-type controls. This deficit correlated with elevated inflammatory responses and the increased susceptibility of endogenous neural stem cells to necroptosis. Mechanistically, lentiviral-mediated Rictor knockdown in neural stem cells in vitro impaired lysosomal function, leading to heightened sensitivity to tumor necrosis factor-alpha- and lipopolysaccharide-induced necroptosis. Collectively, these findings indicate that Rictor/mTORC2 signaling protects endogenous neural stem cells against receptor-interacting protein kinase 1-mediated necroptosis following spinal cord injury. Consequently, the modulation of intrinsic Rictor activity represents a potential therapeutic strategy to enhance endogenous neural stem cell survival and functional recovery post-spinal cord injury.

## Graphical abstract

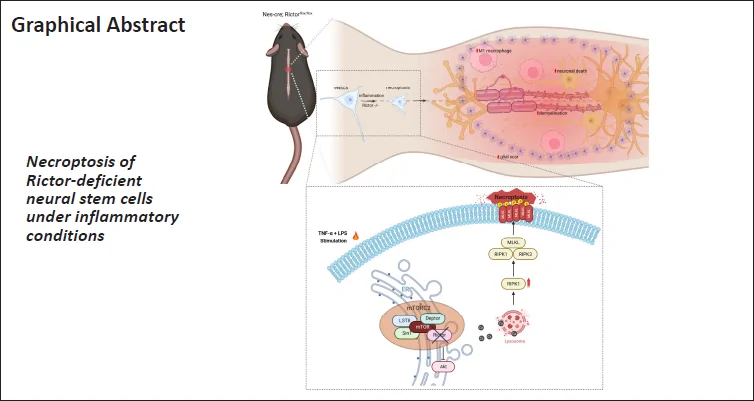

## Introduction

Spinal cord injury (SCI) manifests as a rapid decline or even disappearance of motor and sensory functions, and has high fatality and disability rates (McDonald and Sadowsky, 2002). The complexity of SCI pathogenesis makes effective treatment extremely difficult. To restore functions after SCI, it is crucial to repair the lesion and rebuild neural network connections (Darian-Smith, 2009; Li et al., 2024). Given that stem cells are able to self-renew and differentiate, stem cell therapy has always been a main focus of SCI treatments (Gazdic et al., 2018). However, stem cell therapy has issues related to immunogenicity, efficacy, and safety, which pose important challenges to the treatment of SCI (Cofano et al., 2019; Venkatesh et al., 2019). The discovery of endogenous neural stem cells (eNSCs) with three-lineage differentiation potential in the central nervous system avoids this series of ethical issues and constitutes an important research direction for noninvasive therapy to treat SCI (Sabelström et al., 2014; Hosseini et al., 2024).

In the adult brain, a large number of eNSCs are restricted to the subventricular zone of the lateral ventricle and the subgranular zone of the hippocampal dentate gyrus. In the adult spinal cord, eNSCs mainly line the central canal and are denoted ependymal cells. Weiss et al. (1996) first isolated ependymal cells with self-renewal ability from the adult spinal cord, and reported that these cells are able to differentiate into neurons, oligodendrocytes, and astrocytes. Further studies have demonstrated that prominin-1^+^/forkhead box protein J2^+^/signal transducer CD24^–^ cells in the central canal of the spinal cord exhibit stem cell potential at morphological and genetic levels and play a key role in nerve regeneration (Pfenninger et al., 2011; Alfaro-Cervello et al., 2012). Under normal physiological conditions, eNSCs reside in a dormant state in the ependyma (Sabelström et al., 2013). eNSCs are activated by SCI and exhibit strong proliferation and differentiation ability, migrate from the ependyma to the lesion, and participate in SCI repair (Mothe and Tator, 2005). Activated eNSCs play an important role in glial scar formation, the regulation of neuroinflammation, the reconstruction of neural networks, and neurotrophic support (Hosseini et al., 2024; Tang et al., 2024). However, our previous study revealed that activated eNSCs migrate to the lesion and partially undergo receptor-interacting protein kinase 1 (RIPK1)-dependent necroptosis, which hinders SCI repair (Tong et al., 2023). Promoting the survival of eNSCs and maintaining their function are therefore fundamental to the development of an effective SCI therapy.

**Figure:**
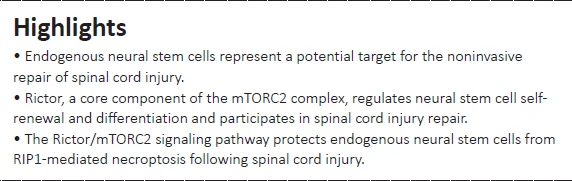

Mammalian target of rapamycin (mTOR) is a highly conserved serine/threonine kinase that forms two complexes: mTORC1 and mTORC2 (Yang et al., 2013). Accumulating evidence suggests that the mTOR signaling pathway drives nerve regeneration (Maiese, 2014; Dhaliwal et al., 2026). mTORC1 is crucial to a variety of cellular processes that are critical for metabolism and growth via its regulation of the phosphorylation of two key downstream initiation factors: S6 kinase and eukaryotic translation initiation factor 4E-binding protein (Goul et al., 2023). In contrast to mTORC1, mTORC2 functions upstream of protein kinase B (AKT), protein kinase C, and serum glucocorticoid-inducible kinase, which play key roles in proliferation, survival, and cytoskeletal remodeling; however, the mechanism by which mTORC2 is regulated is poorly understood (An et al., 2021). Recent research indicates that rapamycin-insensitive companion of mTOR (Rictor)/mTORC2 exerts differential levels of metabolic control in human embryonic stem cells, mesenchymal stem cells, and neural stem cells (NSCs) (Chu et al., 2022), and the loss of protein mono-ADP-ribosyltransferase PARP3 in NSCs causes reduced astrocytic differentiation through the tight regulation of NADPH oxidase 4-induced reactive oxygen species and mTORC2 activation (Rodriguez-Vargas et al., 2020). These findings suggest that mTORC2 is essential for the phenotypic function of NSCs. Our previous research has demonstrated that knockout of the tumor suppressor gene neurofibromatosis-1 enhances the anti-apoptotic properties of NSCs and facilitates neuronal differentiation by activating the Rictor/mTORC2 signaling pathway, thereby affirming the pivotal role of Rictor/mTORC2 in stem cell differentiation (Chen et al., 2022). Moreover, post-SCI, Rictor overexpression significantly alleviates inflammation and curtails neuronal apoptosis, thus indicating a positive effect of the mTORC2/Rictor pathway on neural cell survival (Chen et al., 2020). However, it remains unclear whether the neuroregenerative effects of Rictor are linked to NSC survival.

In the present study, we created a mouse model with the eNSC-specific deletion of Rictor and investigated the function and mechanisms of Rictor in terms of regulating eNSC survival after SCI.

## Methods

### Animals and spinal cord injury model

All animal procedures were approved by the Ethics Committee of the Seventh Affiliated Hospital of Sun Yat-sen University (Approval No. SYSU-IACUC-2024-000274) on February 23, 2024. All experiments were designed and reported according to the Animal Research: Reporting of *In Vivo* Experiments (ARRIVE) guidelines (Percie du Sert et al., 2020). All mice (female, 8–10 weeks old) were housed under controlled environmental conditions (temperature: 24 ± 2°C; humidity: 55% ± 5%; 12-hour light/dark cycle). The homozygous *Nes-Cre;Rictor*^*flox/flox*^ mice and mouse production strategies used in the experiment were provided by Guangdong Yaokang Company (Stock No. B008627, Foshan, Guangdong, China). B6.Cg-Tg(Nes-Cre)1Kln/J transgenic mice (Stock No. 003771) were purchased from Guangdong Yaokang Company for specific knockout of the Rictor gene. The Nes-Cre transgenic mice expressed Cre recombinase in nestin-positive cells in nerve tissue. *Nes-Cre;Rictor*^*flox/flox*^ mice were generated by mating Rictor floxed mice with Nes-Cre transgenic mice, and wild-type (WT) mice (Guangdong Yaokang Company, license No. SCXK (Yue) 2023-0067) were used as controls. DNA was extracted from mouse tails and genotyped using polymerase chain reaction. The primers used for typing were as follows: *Cre* transgene mice (common sequence: 5′-GCC TTA TTG TGG AAG GAC TG-3′, transgene forward sequence: 5′-CCT TCC TGA AGC AGT AGA GCA-3′); *Rictor* genotyping mice (JS04663-Rictor-5wttF1: 5′-CAC ATA GTG CTT TTA ACC TGC CTG G-3′, JS04663-Rictor-5wttR1: 5′-AAC GAA CTT CAC AGA CCT GTC TTC TG-3′).

**Figure 1:**
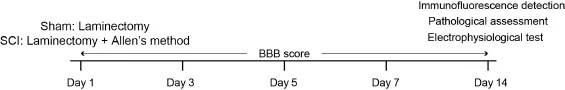
Animal experiment timeline. BBB: Basso, Beattie and Bresnahan; SCI: spinal cord injury.

The SCI model was constructed according to Allen’s method (Allen, 1911). Mice were anesthetized by inhalation of 3% isoflurane (RWD, Shenzhen, Guangdong, China, R510-22) and placed on a platform; anesthesia was maintained by continuous inhalation of 1.8% isoflurane. Laminectomy was performed on the T10 vertebral body of the mouse to fully expose the spinal cord, and then a 10 g hammer was used to hit the spinal cord from a height of 4 cm to induce SCI (Wu et al., 2022). In the sham group, only laminectomy was performed. In the SCI group, Allen’s method was performed following laminectomy. *Nes-Cre;Rictor*^*flox/flox*^ and WT mice were randomly divided into the sham and SCI groups (*n* = 5). Assisted urination was performed once daily after surgery until the voiding reflex recovered. The animal experiment timeline is shown in **Figure 1**.

### Tissue processing

Mice were sacrificed 14 days after the sham or SCI procedure. After being administered inhalational anesthesia (3% isoflurane, RWD), mice were perfused with 10 mL of phosphate-buffered saline (PBS) and 10 mL of 4% paraformaldehyde. Spinal cord tissue containing the injured area was obtained and stored in 4% paraformaldehyde at 4°C. The spinal cord tissue was removed from the 4% paraformaldehyde, washed once with PBS, dehydrated in 15% sucrose for 24 hours, and then placed in 30% sucrose for another 24 hours. Next, the dehydrated spinal cord tissue was embedded in optimal cutting temperature compound and frozen into blocks at −80°C. The blocks were then sectioned (8 μm) using a cryostat (Leica CM1850, Wetzlar, Germany), and the frozen sections were stored at −20°C.

### Immunofluorescence staining

Frozen tissue sections were immersed in PBS for 5 minutes, fixed in 4% paraformaldehyde for 10 minutes, and washed twice with PBS. Triton X-100 (3%) was then used to perforate the membrane for 20 minutes, after which the tissues were washed twice with PBS and blocked with 5% bovine serum albumin for 30 minutes. Subsequently, the sections were incubated overnight at 4°C with the following primary antibodies: anti-RIPK1 antibody (1:250, mouse immunoglobulin G [IgG], BD, Franklin Lakes, NJ, USA, Cat# 610459, RRID: AB_397832), anti-RIPK1 antibody (1:100, rabbit IgG, CST, Boston, MA, USA, Cat# 3493, RRID: AB_2305314), anti-phospho (p)-mixed lineage kinase domain-like protein (MLKL) antibody (1:100, rabbit IgG, CST, Cat# 37333, RRID: AB_2799112), anti-nestin antibody (1:250, rabbit/mouse IgG, Abcam, Cambridge, UK, Cat# ab7659, RRID: AB_2298388 or Cat# ab6142, RRID: AB_305313), anti-Rictor antibody (1:100, rabbit IgG, CST, Cat# 2114, RRID: AB_2179963), anti-CD68 antibody (1:100, rabbit IgG, CST, Cat# 97778, RRID: AB_2928056), anti-arginase 1 (ARG1) antibody (1:100, rabbit IgG, CST, Cat# 93668, RRID: AB_2800207), anti-inducible nitric oxide synthase (iNOS) antibody (1:100, rabbit IgG, CST, Cat# 13120, RRID: AB_2687529), anti-neurofilament-H (NF200) antibody (1:400, mouse IgG, CST, Cat# 2836, RRID:AB_10694081), anti-glial fibrillary acidic protein (GFAP) antibody (1:500, rabbit/mouse IgG, CST, Cat# 80788, RRID: AB_2799963 or Cat# 3670, RRID:AB_561049), anti-gasdermin D (GSDMD) antibody (1:100, mouse IgG, Proteintech, Wuhan, Hubei, China, Cat# 66387-1-Ig, RRID: AB_2881763), anti-cathepsin D (CTSD) antibody (1:100, rabbit IgG, Abcam, Cat# ab75852, RRID: AB_1523267), anti-lysosomal-associated membrane protein 2B (LAMP2B) antibody (1:100, rabbit IgG, Abcam, Cat# ab18529, RRID: AB_2134632), anti-NeuN antibody (1:100, rabbit IgG, CST, Cat# 24307, RRID: AB_2651140), and anti-SOX2 antibody (1:100, mouse IgG, Abcam, Cat# ab79351, RRID: AB_10710406). The sections were then washed three times with PBS before being incubated with secondary antibodies conjugated with 488 or 594 fluorescent compounds (all 1:500, rabbit/mouse IgG, Invitrogen, Carlsbad, CA, USA, Cat# A-11034, RRID: AB_2576217 and Cat# A-11005, RRID: AB_2534073) according to the different host sources at room temperature (26 ± 2°C) for 1 hour. Secondary antibodies were removed, the samples were washed three times with PBS, and nuclei were stained with 4′,6-diamidino-2-phenylindole-containing histology mounting medium (Sigma, St. Louis, MO, USA, F6057). Fluorescence was observed, and images were acquired.

### Immunohistochemical staining

Frozen tissue sections were soaked in PBS for 5 minutes, immersed in 3% hydrogen peroxide in formaldehyde for 20 minutes in the dark, and washed twice with PBS. Next, the sections were immersed in preheated 65°C sodium citrate for 20 minutes for antigen retrieval. After rewarming for 20 minutes, the sections were blocked with goat serum for 30 minutes. They were then incubated with primary antibodies (anti-Rictor antibody, 1:100) overnight at 4°C before being washed three times with PBS, incubated with secondary antibodies (horseradish peroxidase, rabbit IgG, 1:100, CST, Cat# 8114, RRID: AB_10544930) from different genera at room temperature for 1 hour, and washed three times with PBS. After developing the sections with 3,3′-diaminobenzidine, the sections were rinsed with water for 10 minutes. The nuclei were stained with hematoxylin for 2 minutes, differentiated with 1% hydrochloric acid ethanol, and washed with running water for 10 minutes. Finally, the sections were dehydrated, cleared, mounted, and imaged.

### Nissl staining

Frozen tissue sections were immersed in PBS for 5 minutes, fixed in 4% paraformaldehyde for 10 minutes, and washed twice with PBS. Sections were then incubated with Nissl staining solution (Beyotime, Taizhou, Zhejiang, China, C0117) at room temperature for 5 minutes, washed twice with PBS, and immersed in 95% ethanol for 5 seconds. Finally, the sections were dehydrated, cleared, mounted, and imaged. Quantitative assessments of Nissl were conducted using Image Pro Plus software (version ipwin32, Media Cybernetics, Bethesda, MD, USA). This involved the analysis of three consecutive horizontal sections of the spinal cord for each experimental group. For coronal sections of the spinal cord, the software was used to measure the largest area of injury. Subsequently, the number of Nissl bodies within an equivalent area was calculated for the other groups. Statistical analysis was then performed on these measurements.

### Hematoxylin and eosin staining

Frozen tissue sections were immersed in PBS for 5 minutes, fixed in 4% paraformaldehyde for 10 minutes, and washed twice with PBS. The sections were then incubated with hematoxylin staining solution (Beyotime, C0105S) at room temperature for 10 minutes, differentiated with 1% hydrochloric acid ethanol for 3 seconds, and washed with running water for 10 minutes. Next, the sections were incubated with eosin staining solution for 2 minutes and washed with running water for 5 minutes. Finally, the sections were dehydrated, cleared, mounted, and imaged.

### Luxol fast blue staining 

Frozen tissue sections were immersed in PBS for 5 minutes and 95% ethanol for 30 seconds. The sections were then incubated overnight at room temperature in Luxol fast blue staining solution (G-CLONE, Beijing, China, RS1720). Excess staining solution was washed off with 95% ethanol before the sections were washed with running water for 5 minutes. Next, Luxol differentiation solution was used, and 70% ethanol was used to remove the color until the gray and white matter was clear. The sections were then washed with running water for 5 minutes. Finally, the sections were dehydrated, cleared, mounted, and imaged.

### Terminal deoxynucleotidyl transferase dUTP nick end staining

Frozen tissue sections were immersed in PBS for 5 minutes, fixed in 4% paraformaldehyde for 10 minutes, and washed twice with PBS. Triton X-100 (3%) was then used to perforate the membrane for 20 minutes, after which the tissue was washed twice with PBS. Next, the sections were incubated with terminal deoxynucleotidyl transferase dUTP nick end (TUNEL) reagent (Meilunbio, Dalian, Liaoning, China, MA0223) at room temperature for 1 hour. The TUNEL reagent was removed, the sections were washed three times with PBS, and the nuclei were stained with 4′,6-diamidino-2-phenylindole-containing histology mounting medium. Fluorescence was observed, and images were acquired.

### Image acquisition

Fluorescence images were primarily obtained using the confocal Zeiss LSM880 Airyscan (Zeiss, Oberkochen, Germany), Leica DMi8/DM6B microscope (Leica), OLYMPUS APX100 multifunctional microscope (OLYMPUS, Tokyo, Japan), and 3DHISTECH DX12 scanner (3DHISTECH, Jinan, Shandong, China). Photomicrographs of frozen tissue sections stained for pathology (bright field), including Nissl, hematoxylin and eosin, and Luxol fast blue staining, were chiefly acquired using the OLYMPUS APX100 multifunctional microscope and the 3DHISTECH DX12 scanner.

### Enzyme-linked immunosorbent assay

Blood was collected by cardiac puncture before the mice were sacrificed. The blood was then maintained at room temperature for 30 minutes to coagulate. Next, it was centrifuged at 1000 × *g* at 4°C for 15 minutes, and the supernatant serum was collected and stored at −20°C. Serum levels of the inflammatory mediators interleukin-1 beta (IL-1β; Beyotime, P5898) and tumor necrosis factor-alpha (TNF-α; Beyotime, P6020) were detected using enzyme-linked immunosorbent assay kits (Beyotime, PI301, PT512) according to the manufacturer’s instructions. Absorbance was measured at 450 and 630 nm using a BioTek Synergy H1 microplate reader (BioTek, Winooski, VT, USA).

### Behavioral evaluation

The Basso, Beattie, and Bresnahan (BBB) locomotor scale was used for mouse behavioral testing (Basso et al., 1995). The BBB scale quantifies hindlimb motor function; a score of 0 indicates complete paralysis and a score of 21 represents normal locomotion. All functional scores were obtained by two researchers who were blinded to the experimental conditions.

### Electrophysiology

Motor evoked potentials were recorded at 14 days post-SCI to assess neuronal connectivity across the lesion site. Mice were anesthetized using an intraperitoneal injection of compound anesthesia and fixed in a stereotaxic apparatus (Chengdu Instrument Factory, Chengdu, China). After longitudinal scalp incision, a craniotomy was performed to expose the primary motor cortex. A bipolar stimulation electrode (Chengdu Instrument Factory) was stereotaxically positioned below the cortical surface to target corticospinal neurons. A recording needle electrode was inserted into the gastrocnemius muscle ipsilateral to the stimulation site, with a ground electrode attached to the tail. Electrical stimulation was delivered using single square-wave pulses (0.5 mA intensity, 0.5 ms duration, and 1 Hz frequency) via an isolated stimulator. Amplitude was calculated from the onset of the initial response wave to its peak, and all potentials were amplified and recorded using a digital oscilloscope (Chengdu Instrument Factory) (Liu et al., 2024).

### Neural stem cell culture

NSCs were isolated from the spinal cords of embryonic day (E)16–18 C57BL/6 mice (Guangdong Yaokang Company). Briefly, pregnant mice were administered inhalational anesthesia (3% isoflurane, RWD) and sacrificed, the embryos were harvested, and spinal cord tissue was dissected from the embryos. The dissected spinal cord tissue was then cut into small pieces and digested in TrypLE express enzyme (Gibco, Waltham, MA, USA, 12605010) at 37°C for 30 minutes. Next, the digested tissue was centrifuged to remove the enzyme solution. The cells were resuspended in PBS, and the cell suspension was passed through a 70-μm cell sieve and centrifuged again to collect the primary cells. NSCs were cultured in serum-free Dulbecco’s modified Eagle medium/nutrient mixture F-12 (Gibco, 12660012) supplemented with basic fibroblast growth factor (20 ng/mL, Peprotech, Rocky Hill, NJ, USA), epidermal growth factor (20 ng/mL, Peprotech), and 2% B27 supplement. The suspended cells were NSCs and were able to be grown into suspended neurospheres. For subculture or differentiation experiments, the neurospheres were enzymatically dissociated into single cells using Accutase (Gibco, A1110501). The dissociated single NSCs were subsequently plated onto polylysine-coated six-well plates (seeding density: 2 × 10^5^ cells per well) and 12-well plates (seeding density: 1 × 10^5^ cells per well).

**Figure 2:**
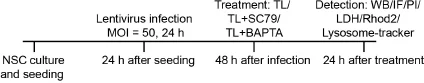
Cell experiment timeline. IF: Immunofluorescence; LDH: lactate dehydrogenase release assay; LPS: lipopolysaccharide; MOI: multiplicity of infection; NSC: neural stem cell; PI: propidium iodide; TL: TNF-α + LPS; TNF-α: tumor necrosis factor-alpha; WB: western blot analysis.

After allowing the plated cells to adhere and grow for at least 24 hours (and typically when cell confluency exceeded 80%), treatments were applied based on specific experimental requirements. For inflammatory stimulation, cells were co-treated with lipopolysaccharide (LPS, 100 ng/mL) and TNF-α (100 ng/mL) for an additional 24 hours (Rong et al., 2019). For the blockade experiment, BAPTA (1 μM, Selleck, Houston, TX, USA, E8148), SC79 (8 μg/mL, Selleck, S7863), and chloroquine (CQ; 10 μM, Selleck, S6999) were added simultaneously with the inflammatory stimulus drug (Wie et al., 2001; Jo et al., 2012; Mauthe et al., 2018). The cell experiment timeline is shown in **Figure 2**.

### Lentivirus construction and neural stem cell transfection

Lentiviral *Rictor*-short hairpin RNA (shRNA) was purchased from Genechem (Shanghai, China) and used to knock down *Rictor* *in vitro*. The sequence information for each primer used to construct the lentivirus was as follows: shC (vector): 5′-CGC TTC CGC GGC CCG TTC AA-3′; shRictor: 5′-ATA GCG CAG CGC TCG CAA CC-3′. NSCs were infected by applying viral supernatant (multiplicity of infection = 50) for 24 hours, after which the medium was replaced with fresh complete medium, and culture was continued.

### Western blot analysis

Total protein was extracted from lysed cells using radioimmune precipitation assay buffer (89901, Thermo Fisher Scientific, Waltham, MA, USA) containing a protease and phosphatase inhibitor cocktail. Total protein (20 or 40 µg) was detected. Proteins were separated by polyacrylamide gel electrophoresis (BN2011BX10, Thermo Fisher Scientific) and transferred to polyvinylidene fluoride membranes (IB24001, Thermo Fisher Scientific) with an iBlot 2 Dry Blotting System (Thermo Fisher Scientific). The blot was then incubated with primary antibodies overnight at 4°C or for 1 hour at room temperature before being washed three times with Tris-buffered saline with Tween. The primary antibodies were anti-RIPK1 antibody (1:1000, mouse IgG, BD, Cat# 610459, RRID: AB_397832), anti-RIPK1 antibody (1:1000, rabbit IgG, CST, Cat# 3493, RRID: AB_2305314), anti-p-MLKL antibody (1:500, rabbit IgG, CST, Cat# 37333, RRID: AB_2799112), anti-Rictor antibody (1:500, rabbit IgG, CST, Cat# 2114, RRID: AB_2179963), anti-CTSD antibody (1:1000, rabbit IgG, Abcam, Cat# ab75852, RRID: AB_1523267), anti-LAMP2B antibody (1:1000, rabbit IgG, Abcam, Cat# ab18529, RRID: AB_2134632), anti-AKT antibody (1:1000, rabbit IgG, CST, Cat# 9272, RRID: AB_329827), anti-p-AKT antibody (1:1000, rabbit IgG, CST, Cat# 4060, RRID: AB_2315049), anti-p62 antibody (1:1000, rabbit IgG, CST, Cat# 39749, RRID:AB_2799160), anti-β-actin antibody (1:1000, rabbit IgG, CST, Cat# 4970, RRID:AB_2223172), and anti-glyceraldehyde-3-phosphate dehydrogenase antibody (1:1000, rabbit IgG, CST, Cat# 5174, RRID:AB_10622025). The blot was then incubated with rabbit-/mouse-specific secondary antibodies (1:1000, rabbit/mouse IgG, CST, Cat# 7074, RRID: AB_2099233 or Cat# 7076, RRID: AB_330924) for 1 hour at room temperature before being washed three times with Tris-buffered saline with Tween. Finally, the protein bands were detected using enhanced chemiluminescence (Epizyme, Cambridge, MA, USA, SQ201) and images were acquired using a Bio-Rad ChemiDoc MP imaging system (Bio-Rad, Hercules, CA, USA).

### Propidium iodide/Hoechst staining

Cells were cultured according to the experimental requirements. The staining working solution was configured according to the Hoechst 33342/propidium iodide (PI) Double Stain kit (Solarbio, Beijing, China). At the end of the cell experiment, the medium was removed, the cells were washed with PBS, an appropriate amount of staining working solution was added, and the cells were incubated at 4°C for 20 minutes. After staining, the cells were washed with PBS once before being observed under a fluorescence microscope (Leica DM6B/DMI8).

### Lactate dehydrogenase release assay

Cells were cultured according to the experimental requirements. At the end of the cell experiment, 120 µL of supernatant was pipetted into a 96-well plate, and 60 µL of lactate dehydrogenase (LDH) detection solution (Beyotime, C0016) was added to each well. The cells were incubated at room temperature for 30 minutes in the dark. Absorbance was measured at 490 nm using a BioTek Synergy H1 microplate reader.

### Lysosome tracking

Cells were cultured according to the experimental requirements. At the end of the cell experiment, the cell culture medium was changed to fresh medium, LysoTracker Red (Beyotime, C1046) was added at a ratio of 1:20,000, and the cells were incubated at 37°C in 5% CO_2_ for 15 minutes. The cells were then quickly observed and imaged under a fluorescence microscope.

### Rhod2 staining

Cells were cultured according to the experimental requirements. At the end of the cell experiment, the medium was removed and cells were washed three times with Ca^2+^- and Mg^2+^-free PBS. Subsequently, Rhod2/AM working solution (YEASEN, Shanghai, China, 40776ES50) was added, and the cells were incubated at 37°C in 5% CO_2_ for 15 minutes. The Rhod2/AM working solution was then removed, and the cells were washed three times with Ca^2+^- and Mg^2+^-free PBS before being incubated in the cell incubator for 20 minutes. Finally, the cells were quickly observed and imaged under a fluorescence microscope.

### Statistical analysis

All statistical tests were performed using GraphPad Prism 8 software (GraphPad Software, Boston, MA, USA, www.graphpad.com). Data normality was assessed using the Shapiro–Wilk test, and homogeneity of variance was confirmed using Levene’s test. For datasets conforming to both normality and equal variance assumptions, one-way analysis of variance was performed, followed by Bonferroni *post hoc* tests for multiple comparisons if the analysis of variance was significant (*P* < 0.05). Two-way repeated measures analysis of variance was conducted to assess the effects of group and time on BBB scores, followed by the Bonferroni correction for multiple comparisons. All values are presented as the mean ± standard deviation (SD), and *P*-values were considered significant at < 0.05.

## Results

### Generation of mice with endogenous neural stem cell–specific deletion of Rictor

**Figure 3:**
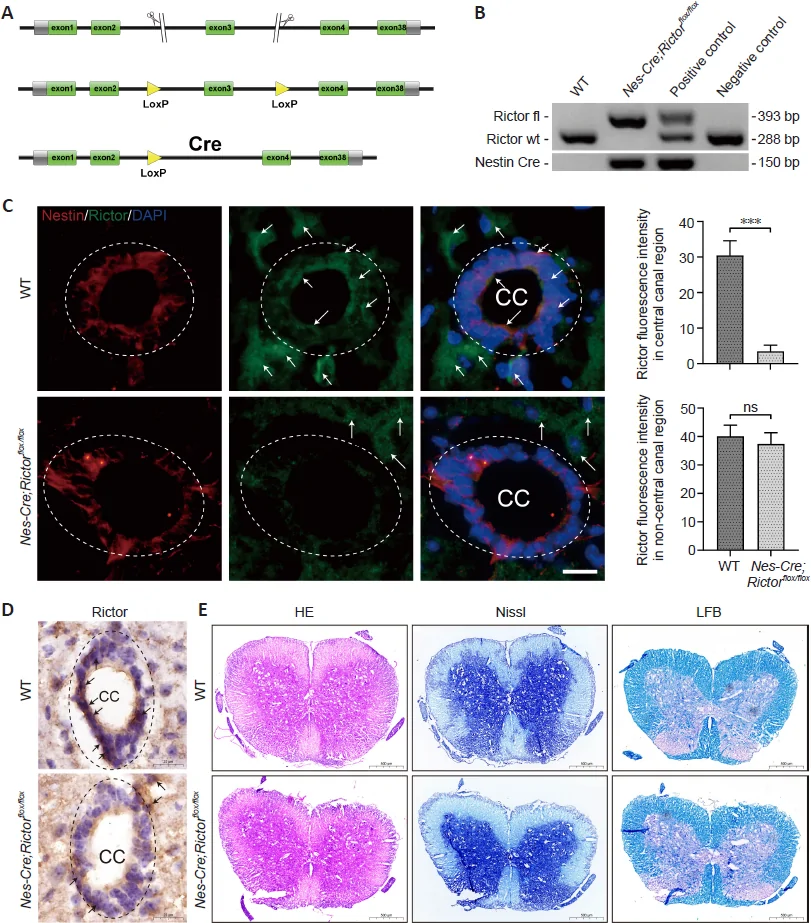
Generation of mice with the eNSC-specific deletion of Rictor. (A) Strategy for generating mice with the eNSC-specific deletion of Rictor. Created using Adobe Illustrator 2023. (B) Genotyping of the mice was performed by polymerase chain reaction analysis of genomic DNA. (C) Immunofluorescence analysis of Rictor expression in eNSCs within the CC. eNSCs were labeled with nestin (red, Alexa Fluor 594). In WT mice, nestin and Rictor (green, Alexa Fluor 488) co-localized in CC eNSCs. In *Nes-Cre;Rictor*^*flox/flox*^ mice, nestin expression persisted in eNSCs, whereas Rictor expression was significantly reduced specifically within the CC. Rictor expression outside the CC area showed no significant difference between genotypes. Quantification of Rictor immunofluorescence intensity (*n* = 3). Rictor signal was significantly lower in the CC but not outside the CC in *Nes-Cre;Rictor*^*flox/flox*^ mice compared with that in the WT group. The dashed box represents the CC region. The white arrow indicates the Rictor-positive fluorescence area. All data are presented as the mean ± SD. ***P < 0.001 (two-tailed unpaired Student’s *t*-test). Scale bar: 10 μm. (D) Histochemical analysis of Rictor expression in the CC region. Immunohistochemical staining revealed significantly reduced Rictor expression specifically in the CC region of *Nes-Cre;Rictor*^*flox/flox*^ mice compared with that in WT mice. The dashed box represents the CC region. The black arrow indicates the Rictor-positive fluorescence area. Scale bar: 20 μm. (E) Pathology of the mouse spinal cord according to HE, Nissl, and LFB staining. No significant difference was observed in the pathological tissues between the two groups. Scale bars: 500 μm. CC: Central canal; eNSC: endogenous neural stem cell; HE: hematoxylin and eosin; ns: not significant; LFB: Luxol Fast Blue; SCI: spinal cord injury; WT: wild-type.

Rictor is a key component protein of the mTORC2 complex and plays a pivotal role in regulating the mTORC2 signaling pathway (Sarbassov et al., 2004). To explore the role of Rictor/mTORC2 in NSCs, we used the *Cre-LoxP* system to generate conditional knockout mice with a specific deletion of the *Rictor* gene in nestin-positive NSCs in the central canal (**Figure 3A**). Polymerase chain reaction analysis demonstrated the correct targeting of ependymal nestin-positive NSCs and gene-targeted mice (**Figure 3B**). We then assessed Rictor expression in the central canal eNSCs of the spinal cord using immunofluorescence and immunohistochemical staining. Rictor expression in the central canal was significantly lower in *Nes-Cre;Rictor*^*flox/flox*^ mice than in WT mice (**Figure 3C** and **D**). The *Nes-Cre;Rictor*^*flox/flox*^ mice were born and developed normally. We performed histological assessments of the spinal cords at 8 weeks after birth. No significant differences in histology were observed between the two groups using hematoxylin and eosin, Nissl, or Luxol fast blue staining (**Figure 3E**). Together, these results suggest that the absence of Rictor in spinal cord eNSCs does not affect the growth and development of mice under normal physiological conditions.

### Endogenous neural stem cell–specific deletion of Rictor weakens functional recovery after spinal cord injury in mice

**Figure 4:**
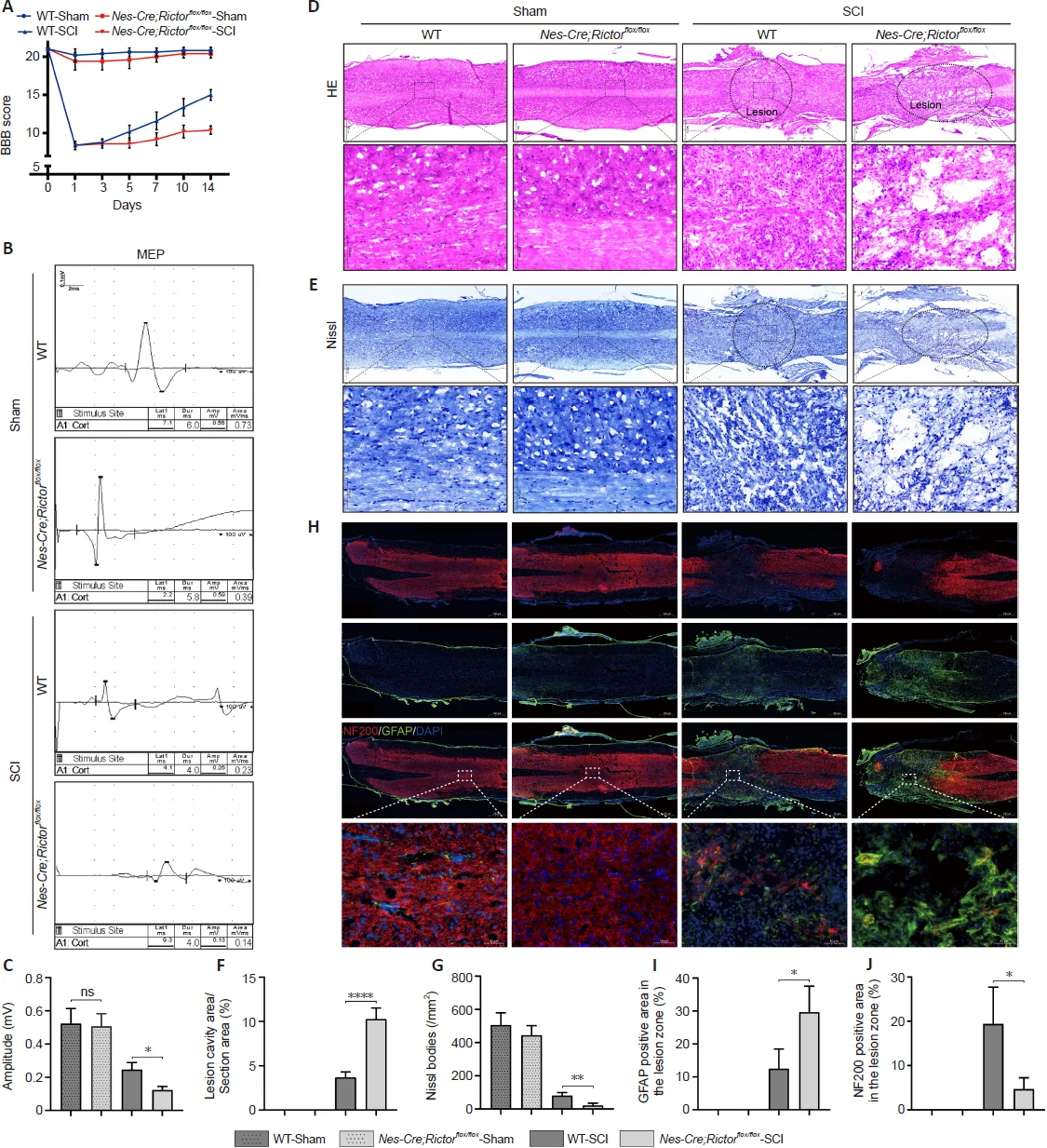
eNSC-specific deletion of *Rictor* weakens functional recovery after SCI in mice. (A) BBB limb function scores at 14 days post-SCI. Higher scores indicate better motor functional recovery (two-way repeated measures analysis of variance followed by the Bonferroni correction for multiple comparisons). (B, C) Electrophysiological assessment of MEPs at 14 days post-SCI. Quantification of MEP amplitude in mice. *Nes-Cre;Rictor*^*flox/flox*^ mice exhibited significantly reduced MEP amplitude compared with WT mice in the SCI group, whereas no inter-genotypic differences were observed in the sham group. (D, E) Histopathological analysis of the mouse spinal cord using hematoxylin and eosin staining and Nissl staining. *Nes-Cre;Rictor*^*flox/flox*^ SCI mice showed exacerbated tissue disorganization and structural disruption compared with WT SCI mice. The sham groups showed comparable histoarchitecture. Scale bars: 500 μm (upper), 100 μm (lower). (F) Quantification of the lesion cavity area in the spinal cord sections, which reflects the extent of post-traumatic tissue cavitation. (G) Quantification of Nissl bodies in the spinal cord sections. Nissl staining highlighting the neuronal cell bodies. The number and distribution of Nissl bodies can indicate the health and density of neurons. (H) Immunofluorescence co-staining of an axonal marker (NF200, red, Alexa Fluor 594) and an astroglial marker (GFAP, green, Alexa Fluor 488) in the mouse spinal cord at 14 days post-SCI. Scale bars: 500 μm, 50 μm (enlarged images). (I, J) Quantitative analysis of the GFAP^+^ and NF200^+^ immunofluorescence area in the lesion. The GFAP^+^ area was significantly expanded in the *Nes-Cre;Rictor*^*flox/flox*^ SCI mice. The NF200^+^ area was significantly smaller in *Nes-Cre;Rictor*^*flox/flox*^ mice than in WT mice at 14 days post-SCI. All data are presented as the mean ± SD (*n* ≥ 3). *P < 0.05, **P < 0.01, ****P < 0.0001 (two-way repeated measures analysis of variance followed by the Bonferroni correction for multiple comparisons [A] or one-way analysis of variance followed by the Bonferroni correction for multiple comparisons [C, G–J]). BBB: Basso, Beattie and Bresnahan; eNSC: endogenous neural stem cell; GFAP: glial fibrillary acidic protein; HE: hematoxylin and eosin; MEP: motor evoked potential; NF200: neurofilament-H; ns: not significant; SCI: spinal cord injury; WT: wild-type.

Recent research indicates that Rictor plays an important role in the self-renewal and differentiation of stem cells (Chu et al., 2022). Our previous study demonstrated that Rictor overexpression can promote functional recovery after SCI (Chen et al., 2020). To further determine the role of Rictor in eNSCs in SCI repair, we established an SCI mouse model using Allen’s method. In this method, incomplete damage to the spinal cords of mice is induced using appropriate blows, and the motor functions of the mice gradually recover after SCI (Wu et al., 2022). The BBB score was used to evaluate the motor ability of mice after SCI. There was no difference in BBB scores between WT and *Nes-Cre;Rictor*^*flox/flox*^ mice in the sham group; however, in the SCI group, the BBB scores of *Nes-Cre;Rictor*^*flox/flox*^ mice were significantly lower than those of WT mice from day 3 after SCI (**Figure 4A**). We also performed electrophysiological tests on the mice at 14 days after SCI. In the SCI group, the potential amplitude of *Nes-Cre;Rictor*^*flox/flox*^ mice was significantly lower than that of WT mice (**Figure 4B** and **C**). These results indicate that the ablation of Rictor in eNSCs may hinder motor recovery after SCI. Furthermore, we performed hematoxylin and eosin and Nissl staining of the spinal cord. Compared with the sham group, the SCI group exhibited clear structural disorder (**Figure 4D** and **E**). In the SCI group, *Nes-Cre;Rictor*^*flox/flox*^ mice exhibited more and larger cavities in the lesion and fewer Nissl bodies compared with WT mice (**Figure 4D–G**). Similarly, the number of NeuN-positive neurons in *Nes-Cre;Rictor*^*flox/flox*^ mice by immunofluorescence staining was also significantly reduced (**Additional Figure 1A** and **B**). These results suggest that the ablation of Rictor in eNSCs affects spinal cord tissue repair after SCI.

Previous studies indicate that glial scar tissue forms around the center of the lesion after SCI (Fitch and Silver, 2008; Clifford et al., 2023). Activated astrocytes are the major cells constituting glial scars. Although the function of glial scar formation after SCI remains controversial, most studies have demonstrated that it represents the main hindrance to axonal regeneration in the subacute and chronic injury stages (Tran et al., 2022). In the present study, we examined the formation of glial scars after SCI. In the sham group, GFAP (the main marker of astrocytes) was expressed sporadically in the spinal cord (**Figure 4H**). After SCI, a relatively large amount of GFAP expression was detected in the lesion, suggesting that astrocytes were activated and gathered in the injured area to participate in the repair of spinal cord tissue (**Figure 4H**). Compared with that of the WT SCI group, the GFAP-positive lesion area of the *Nes-Cre;Rictor*^*flox/flox*^ group was significantly larger (**Figure 4I**). We also detected NF200, which is an important marker of axonal regeneration. Compared with that in WT mice, the NF200-positive area in the lesion was significantly lower in *Nes-Cre;Rictor*^*flox/flox*^ mice after SCI (**Figure 4H** and **J**). These results suggest that the ablation of Rictor in eNSCs leads to larger glial scars after SCI, and that the enlarged glial scars may impede axonal regeneration.

### Endogenous neural stem cell–specific deletion of Rictor exacerbates inflammatory cell infiltration in lesions after spinal cord injury

**Figure 5:**
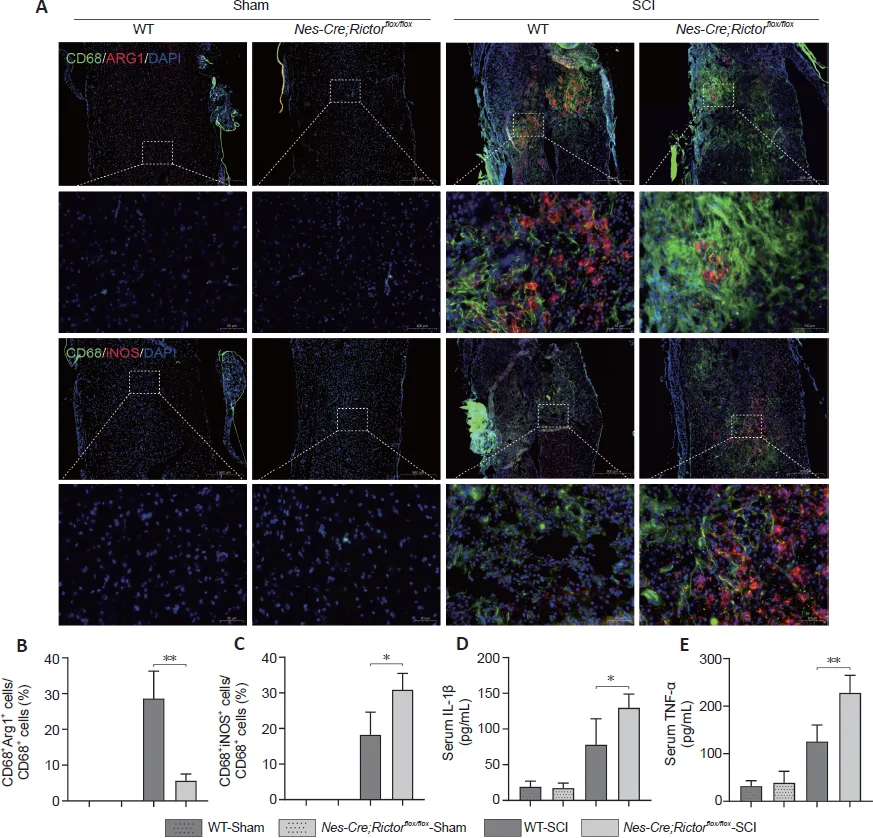
eNSC-specific deletion of Rictor amplifies neuroinflammation post-SCI. (A) Immunofluorescence staining showing macrophages/activated microglia labeled with CD68 (green, Alexa Fluor 488). Polarization was detected using the M2 marker ARG1 (red, Alexa Fluor 594) and the M1 marker iNOS (red, Alexa Fluor 594). *Nes-Cre;Rictor*^*flox/flox*^ SCI mice exhibited enhanced iNOS^+^ staining but attenuated ARG1^+^ signal *versus* WT SCI mice. Scale bars: 500 μm, 50 μm (enlarged images). (B, C) Quantitative determination of CD68^+^ARG1^+^ cells and CD68^+^iNOS^+^ cells among the different groups. CD68^+^ARG1^+^ cells were significantly increased in *Nes-Cre;Rictor*^*flox/flox*^ SCI mice, whereas CD68^+^iNOS^+^ cells were significantly decreased. (D, E) Serum levels of IL-1β (D) and TNF-α (E) were measured using ELISA. Serum levels of IL-1β and TNF-α were significantly higher in *Nes-Cre;Rictor*^*flox/flox*^ SCI mice than in WT SCI mice. All data are presented as the mean ± SD (*n* ≥ 3). *P < 0.05, **P < 0.01 (one-way analysis of variance followed by the Bonferroni correction for multiple comparisons). ARG1: Arginase 1; DAPI: 4′,6-diamidino-2-phenylindole; ELISA: enzyme-linked immunosorbent assay; eNSC: endogenous neural stem cell; IL-1β: interleukin-1 beta; iNOS: inducible nitric oxide synthase; ns: not significant; SCI: spinal cord injury; TNF-α: tumor necrosis factor-alpha; WT: wild-type.

The infiltration of inflammatory cells is an important mechanism of secondary SCI, and eNSCs play an important role in the regulation of inflammation after SCI (Rong et al., 2019; Gampierakis et al., 2021). To assess whether the ablation of Rictor in eNSCs affects inflammatory cell infiltration in SCI, we labeled macrophages/activated microglia with CD68 using immunofluorescence staining and identified the M1 and M2 polarization of macrophages/activated microglia with ARG1 and iNOS, respectively. The expression of CD68 in the lesion area was significantly higher in the SCI group than in the sham group (**Figure 5A**). In the SCI group, the number of CD68/iNOS double-positive cells in *Nes-Cre;Rictor*^*flox/flox*^ mice was significantly higher than that in WT mice, whereas the number of CD68/ARG1 double-positive cells was significantly lower (**Figure 5A–C**). In addition, in the SCI group, the serum levels of IL-1β and TNF-α were significantly higher in *Nes-Cre;Rictor*^*flox/flox*^ mice than in WT mice, as detected using enzyme-linked immunosorbent assay analysis (**Figure 5D** and **E**). These findings indicate that the ablation of Rictor in eNSCs increases M1-type polarization of inflammatory cells and serum levels of IL-1β and TNF-α, thereby suggesting an exacerbation of inflammation in *Nes-Cre;Rictor*^*flox/flox*^ mice after SCI.

### Knockdown of *Rictor* promotes neural stem cell necroptosis in lesions after spinal cord injury or inflammatory stimulation

**Figure 6:**
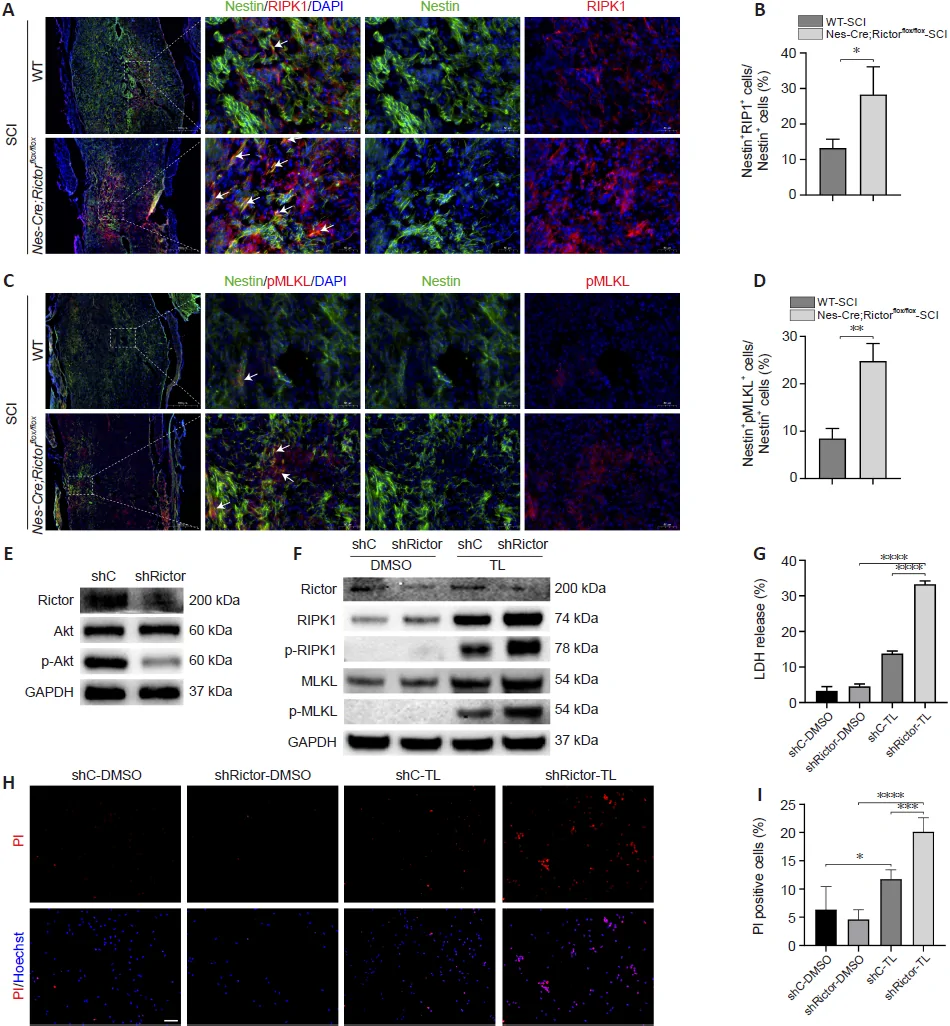
Rictor deficiency sensitizes NSCs to necroptosis. (A, B) *In vivo* necroptosis activation in eNSCs. Representative co-staining of the NSC marker nestin (green, Alexa Fluor 488) and the necroptosis initiator RIPK1 (red, Alexa Fluor 594) at 14 days post-SCI. Quantification of nestin^+^RIPK1^+^ cells. *Nes-Cre;Rictor*^*flox/flox*^ SCI mice exhibited significantly increased double-positive cells compared with WT SCI mice. Scale bars: 500 μm, 50 μm (enlarged images). (C, D) Necroptosis executor phosphorylation *in vivo*. Co-localization of nestin (green, Alexa Fluor 488) and p-MLKL (red, Alexa Fluor 594). Nestin^+^p-MLKL^+^ cells were significantly elevated in *Nes-Cre;Rictor*^*flox/flox*^ SCI. Scale bars: 500 μm, 50 μm (enlarged images). (E) Expression of Rictor, AKT, and p-AKT in NSCs infected with shC or shRictor lentivirus *in vitro*. GAPDH was used as a loading control. (F) Expression of RIPK1, p-RIPK1, MLKL, and p-MLKL in shC-NSCs and shRictor-NSCs after 24 hours of TL treatment. Notably, there were significant increases in RIPK1, p-RIPK1, and p-MLKL in shRictor-NSCs compared with the findings in shC-NSCs after 24 hours of TL treatment. GAPDH was used as a loading control. (G) LDH release in shC-NSCs and shRictor-NSCs after 24 hours of TL treatment. Increased LDH release in shRictor-NSCs post-TL treatment. (H, I) PI/Hoechst staining in shC-NSCs and shRictor-NSCs after 24 hours of TL treatment. Quantified PI^+^ cells showing a significant increase in cell death in shRictor-NSCs post-TL treatment. Scale bar: 100 μm. All data are presented as the mean ± SD (*n* = 3). *P < 0.05, **P < 0.01, ***P < 0.001, ****P < 0.0001 (two-tailed unpaired Student’s *t*-test [B, D] or one-way analysis of variance followed by the Bonferroni correction for multiple comparisons [G, I]). Akt: Protein kinase B; DAPI: 4′,6-diamidino-2-phenylindole; DMSO: dimethyl sulfoxide; eNSC: endogenous neural stem cell; GAPDH: glyceraldehyde-3-phosphate-dehydrogenase; LDH: lactate dehydrogenase; LPS: lipopolysaccharide; MLKL: mixed lineage kinase domain-like protein; NSC: neural stem cell; p-Akt: phospho-protein kinase B; PI: propidium iodide; pMLKL: phospho-mixed lineage kinase domain-like protein; pRIPK1: phospho-receptor interacting protein kinase 1; RIPK1: receptor interacting protein kinase 1; SCI: spinal cord injury; TL: TNF-α + LPS; TNF-α: tumor necrosis factor-alpha; WT: wild-type.

After SCI, the inflammatory response in the center of the lesion often leads to the death of nerve cells (Shi et al., 2021). We previously reported that eNSCs undergo necroptosis in the injured area after SCI (Tong et al., 2023). To investigate whether Rictor affects eNSC survival after SCI, we assessed the survival of eNSCs in SCI lesions. RIPK1 and GSDMD are important proteins that are involved in necroptosis and pyroptosis, respectively, and TUNEL staining can reflect apoptosis (Bertheloot et al., 2021). We detected nestin/RIPK1, nestin/GSDMD, and nestin/TUNEL double immunofluorescence staining at 14 days after SCI. Compared with those in WT mice, the percentages of nestin/RIPK1 double-positive cells were significantly greater in lesions of *Nes-Cre;Rictor*^*flox/flox*^ mice, whereas the percentages of nestin/GSDMD and nestin/TUNEL double-positive cells were not significantly different (**Figure 6A**, **B**, and **Additional Figure 2A–D**). We also detected the expression of p-MLKL (an executive molecule of necroptosis) (Wang et al., 2014). *Nes-Cre;Rictor*^*flox/flox*^ mice had significantly more nestin/p-MLKL double-positive cells than WT mice at 14 days after SCI (**Figure 6C** and **D**). These results suggest that the ablation of Rictor may increase the sensitivity of eNSCs to post-SCI necroptosis.

To clarify whether the ablation of *Rictor* in NSCs sensitizes them to necroptosis, we extracted NSCs from the mouse spinal cord for further *in vitro* experiments (**Additional Figure 3A–C**). First, we used a lentivirus with Rictor shRNA (shRictor) or control shRNA (shC) to infect NSCs, and then detected Rictor expression using western blot analysis. As shown in **Figure 6E**, cells expressing shRictor displayed robust downregulation of Rictor protein and decreased p-AKT (a downstream protein of Rictor) (Betz et al., 2013), indicating that Rictor was knocked down in NSCs and inhibited mTORC2 signaling. Next, we simulated the inflammatory environment *in vitro* by co-treating NSCs with TNF-α and LPS. Under physiological conditions, Rictor knockdown did not lead to the increased expression of RIPK1, p-RIPK1, MLKL, or p-MLKL, suggesting that Rictor knockdown does not induce necroptosis (**Figure 6F**). After TNF-α and LPS treatment, the expression levels of RIPK1, p-RIPK1, MLKL, and p-MLKL in the shC-NSCs were slightly increased, whereas the expression levels of RIPK1, p-RIPK1, MLKL, and p-MLKL in the shRictor-NSCs were significantly increased (**Figure 6F**). We further performed PI staining and LDH release experiments on these cells to identify necroptosis. In the inflammatory environment, the shRictor group had significantly greater numbers of PI-positive cells and greater release of LDH compared with the shC group (**Figure 6G–I**). These results suggest that NSCs with Rictor knockdown are sensitized to necroptosis under inflammatory stimuli.

### Knockdown of *Rictor* impairs the lysosomal function of neural stem cells, leading to RIPK1 accumulation under inflammation

**Figure 7:**
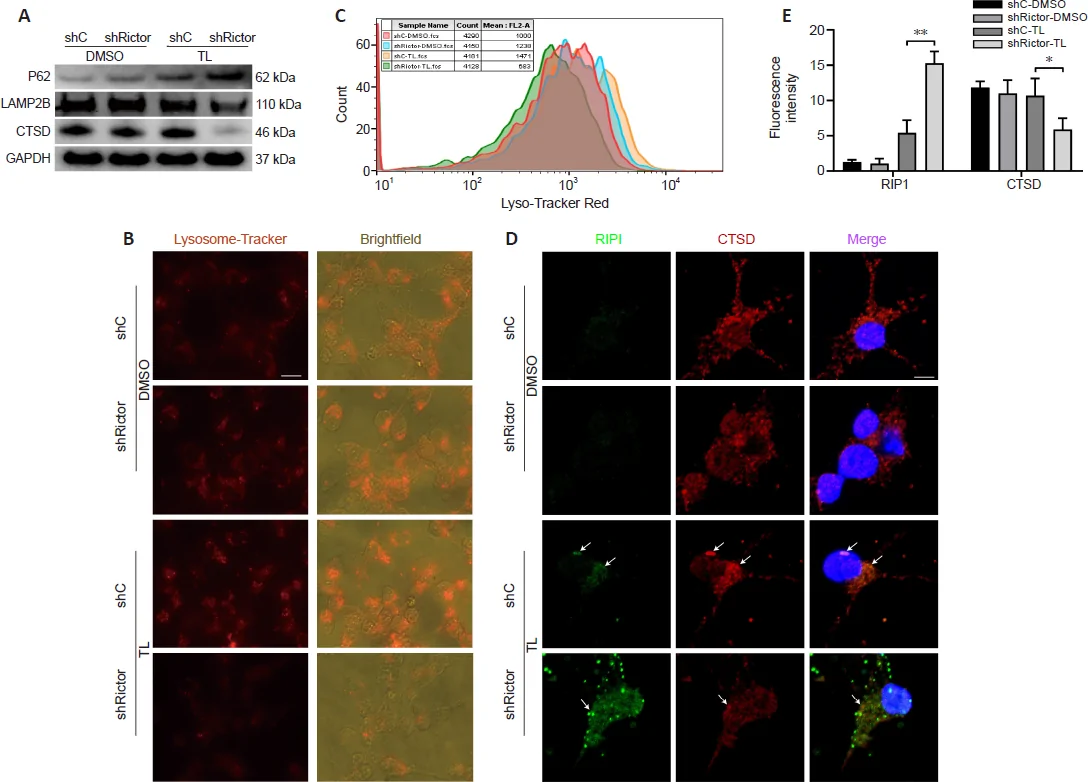
Knockdown of Rictor impairs the lysosomal function of NSCs, leading to RIPK1 accumulation under inflammatory conditions. (A) Western blot of P62, LAMP2B, and CTSD in shC/shRictor-NSCs after 24 hours of TL treatment. GAPDH was used as a loading control. (B) LysoTracker was used to track lysosomes in shC-NSCs and shRictor-NSCs after 24 hours of TL treatment. Rictor knockdown in NSCs under inflammatory conditions led to a reduction in lysosomes. Scale bar: 10 μm. (C) Quantification of LysoTracker fluorescence in shC-NSCs and shRictor-NSCs after 24 hours of TL treatment, assessed using flow cytometry. (D, E) Immunofluorescence co-staining showing the expression of RIPK1 (green, Alexa Fluor 488) and CTSD (red, Alexa Fluor 594) in shC-NSCs and shRictor-NSCs after 24 hours of TL treatment. Yellow puncta indicate co-localization (white arrows). The expression of RIPK1 was significantly increased in shRictor-NSCs after 24 hours of TL treatment, whereas CTSD expression was significantly decreased. Scale bar: 5 μm. All data are presented as the mean ± SD (*n* = 3). *P < 0.05, **P < 0.01 (one-way analysis of variance followed by the Bonferroni correction for multiple comparisons). CTSD: Cathepsin D; DMSO: dimethyl sulfoxide; GAPDH: glyceraldehyde-3-phosphate-dehydrogenase; LAMP2B: lysosomal-associated membrane protein 2B; LPS: lipopolysaccharide; NSC: neural stem cell; RIPK1: receptor interacting protein kinase 1; SCI: spinal cord injury; TL: TNF-α + LPS; TNF-α: tumor necrosis factor-alpha.

Recent studies have highlighted the importance of the lysosomal pathway in central nervous system diseases (Udayar et al., 2022). Lysosomal dysfunction after SCI inhibits autophagy and sensitizes cells to necroptosis by promoting RIPK1 accumulation (Liu et al., 2018). We explored whether the necroptosis sensitization induced by Rictor knockdown in NSCs in an inflammatory environment is related to lysosome function. *Rictor* knockdown significantly increased the protein expression of P62 after TNF-α and LPS treatment (**Figure 7A**). Previous research has revealed that P62 is mainly degraded through lysosomes, and that P62 accumulation suggests that the lysosomal degradation pathway is blocked (Sánchez-Martín et al., 2019). We further examined lysosomal function after TNF-α and LPS treatment. LAMP2B is a marker protein of lysosomal membranes, whereas CTSD is a cathepsin protein in lysosomes (Qiao et al., 2023). After TNF-α and LPS treatment, the expression levels of LAMP2B and CTSD were significantly decreased in shRictor-NSCs, indicating impaired lysosomal function (**Figure 7A**). Similarly, the LysoTracker assessment of cell lysosomes demonstrated that *Rictor* knockdown in NSCs under inflammatory conditions led to a reduction in lysosomes (**Figure 7B** and **C**). Moreover, we confirmed the co-localization of RIPK1 with CTSD and LAMP2B using immunofluorescence staining; the results suggest that RIPK1 can be degraded through the lysosomal pathway (**Figure 7D**, **E**, and **Additional Figure 4**). In NSCs with *Rictor* knockdown, CTSD and LAMP2B fluorescence decreased significantly and RIPK1 fluorescence increased significantly after TNF-α and LPS treatment, consistent with the western blot analysis results (**Figure 7D**, **E**, and **Additional Figure 4**).

The lysosomotropic agent CQ aggregates in acidic lysosomes to raise lysosomal pH, thereby inhibiting lysosomal hydrolases and preventing autophagosome fusion and degradation (Mauthe et al., 2018). To confirm that impaired lysosomal function leads to RIPK1 accumulation, we co-treated NSCs with TNF-α, LPS, and CQ. CQ treatment increased the ratios of microtubule-associated protein 1 light chain 3 A/B (LC3A/B)-I and LC3A/B-II and increased P62 expression (**Additional Figure 5A**), indicating that CQ inhibits the lysosomal pathway. In addition, the protein levels of RIPK1 and p-MLKL were significantly increased after TNF-α, LPS, and CQ co-treatment, further demonstrating that blocking the lysosomal pathway leads to RIPK1 accumulation (**Additional Figure 5A**). These results suggest that under inflammatory conditions, the impairment of lysosomal function in NSCs with Rictor knockdown leads to RIPK1 accumulation and sensitization to necroptosis.

### SC79 and BAPTA-AM inhibit necroptosis in neural stem cells with *Rictor* knockdown under inflammation by reducing intracellular Ca^2+^ levels

**Figure 8:**
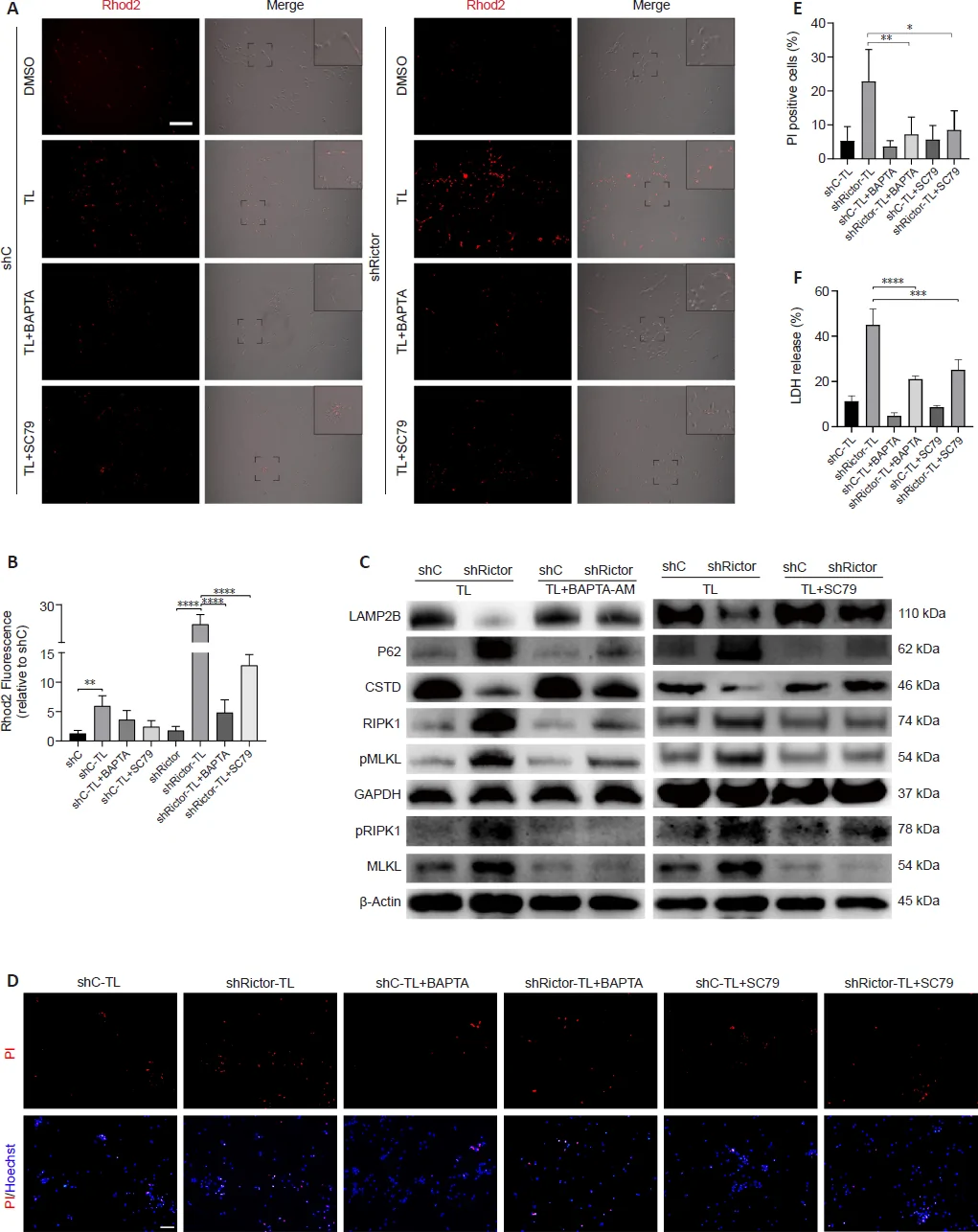
SC79 and BAPTA-AM inhibit necroptosis in NSCs with *Rictor* knockdown under inflammation. (A, B) Fluorescence staining with Rhod2 dye indicates the intracellular Ca^2+^ levels in shC-NSCs and shRictor-NSCs after 24 hours of TL, TL + BAPTA-AM, and TL + SC79 treatment. Quantification of Rhod2 fluorescence in shC-NSCs and shRictor-NSCs after 24 hours of TL, TL + BAPTA-AM, and TL + SC79 treatment. After TL treatment, the intracellular Ca^2+^ of NSCs with *Rictor* knockdown was significantly increased, while BAPTA-AM and SC79 significantly inhibited the aforementioned changes. Scale bar: 100 μm. (C) Western blot analysis of LAMP2B, P62, CTSD, RIPK1, p-RIPK1, MLKL, and p-MLKL expression in shC-NSCs and shRictor-NSCs after 24 hours of treatment with TL, TL + BAPTA-AM, or TL + SC79. GAPDH was used as a loading control. BAPTA-AM and SC79 increased the expression of LAMP2B and CTSD in shRictor-NSCs after 24 hours of TL treatment and decreased the expression of P62, RIPK1, p-RIPK1, MLKL, and p-MLKL. (D, E) PI/Hoechst staining in shC-NSCs and shRictor-NSCs after 24 hours of TL, TL + BAPTA-AM, and TL + SC79 treatment. BAPTA-AM or SC79 reduced the PI^+^ cells in the shRictor-TL group. Scale bar: 100 μm. (F) LDH release assay in shC-NSCs and shRictor-NSCs after 24 hours of treatment. BAPTA-AM and SC79 significantly reduced LDH release in TL-treated shRictor-NSCs. All data are presented as the mean ± SD (*n* = 3). *P < 0.05, **P < 0.01, ***P < 0.001, ****P < 0.0001 (one-way analysis of variance followed by the Bonferroni correction for multiple comparisons). CTSD: Cathepsin D; DMSO: dimethyl sulfoxide; GAPDH: glyceraldehyde-3-phosphate-dehydrogenase; LAMP2B: lysosomal-associated membrane protein 2B; LDH: lactate dehydrogenase; LPS: lipopolysaccharide; MLKL: mixed lineage kinase domain-like protein; NSC: neural stem cell; PI: propidium iodide; pMLKL: phospho-mixed lineage kinase domain-like protein; pRIPK1: phospho-receptor interacting protein kinase 1; RIPK1: receptor interacting protein kinase 1; SCI: spinal cord injury; TL: TNF-α + LPS; TNF-α: tumor necrosis factor-alpha.

Previous studies have reported that Rictor/mTORC2 is located mainly in the endoplasmic reticulum and mitochondria and plays an important role in their homeostasis (Boulbés et al., 2011; Betz et al., 2013). Rictor loss diminishes AKT phosphorylation at S473 in the endoplasmic reticulum membrane, thereby causing an imbalance in endoplasmic reticulum homeostasis and releasing Ca^2+^ (Chen et al., 2011). Intracellular Ca^2+^ levels are closely related to lysosomal function (Lloyd-Evans and Waller-Evans, 2020). To evaluate whether Rictor knockdown damages lysosomes by releasing Ca^2+^, we examined intracellular Ca^2+^ release using the cell-permeable fluorescent Ca^2+^ indicator Rhod2-AM. Rictor knockdown had no effect on intracellular Ca^2+^ under physiological conditions; however, intracellular Ca^2+^ was significantly increased in NSCs with Rictor knockdown after TNF-α and LPS treatment (**Figure 8A** and **B**). Subsequently, treatment with the cell-permeable Ca^2+^ chelator BAPTA-AM and the AKT phosphorylation agonist SC79 significantly reduced intracellular Ca^2+^ in NSCs with Rictor knockdown after TNF-α and LPS treatment, suggesting that intracellular Ca^2+^ release is regulated by the Rictor/mTORC2 signaling pathway (**Figure 8A**, **B**, and **Additional Figure 5B**). Furthermore, western blot analysis revealed that treatment with BAPTA-AM and SC79 increased the expression of LAMP2B and CTSD and decreased the expression of P62 in NSCs with Rictor knockdown under TNF-α and LPS treatment, suggesting the restoration of lysosomal function (**Figure 8C**).

Next, we examined whether BAPTA-AM and SC79 can inhibit the necroptosis induced by Rictor knockdown under TNF-α and LPS treatment. Treatment with BAPTA-AM and SC79 reduced the protein expression of RIPK1, p-RIPK1, MLKL, and p-MLKL; the number of PI-positive cells; and the release of LDH in NSCs with Rictor knockdown under TNF-α and LPS treatment. These findings suggest that the restoration of lysosomal function reduces RIPK1 accumulation and suppresses RIPK1/p-MLKL-mediated necroptosis (**Figure 8C–F**).

## Discussion

In the present study, we investigated whether Rictor deletion exerts protective effects in a mouse model of SCI. We generated a mouse model with eNSC-specific deletion of *Rictor* and demonstrated that endogenous Rictor/mTORC2 has a critical role in repair after SCI that may be mediated via the promotion of eNSC survival through AKT signaling activation and necroptosis inhibition. Notably, the specific deletion of *Rictor* in eNSCs made mice susceptible to necroptosis in the spinal cord lesion and hindered repair after SCI. *In vitro* experiments revealed that the ablation of Rictor reduced RIPK1 degradation by impairing lysosomal function under inflammatory stimuli, which increased the necroptotic sensitivity of NSCs.

SCI has complex pathology that can lead to severe motor and sensory dysfunction (Eckert and Martin, 2017). Stem cell therapy is potentially protective in view of its broad-spectrum efficacy and has been extensively investigated preclinically in different SCI models (Cofano et al., 2019; Shao et al., 2019). In particular, the discovery of eNSCs is promising for the noninvasive treatment of SCI. A rapid increase in the expression of the eNSC marker nestin after SCI indicates that these cells are activated (Grégoire et al., 2015; Rong et al., 2019). On day 3 after SCI, proliferation and migration capabilities reach their peak, and eNSCs are recruited to the lesion to play a key role in its repair (Mothe and Tator, 2005). Although the role of the reactive astrocytes that form the glial scar surrounding the lesion remains controversial, the glial scar plays a positive role in limiting the secondary expansion of the injury and further loss of axons (Adams and Gallo, 2018). More than half of all astrocytes in glial scars are derived from ependymal cells (Sabelström et al., 2013). Ependymal cells with Ras knockout stop proliferating, which prevents the formation of the NSC-derived component of the glial scar. This results in the spread of inflammation, loss of neurons in the lesion, and reduced secretion of neurotrophic factors (Sabelström et al., 2013). Promoting the survival and function of eNSCs is therefore of great importance for SCI repair.

The role of the mTOR signaling pathway in SCI physiology and pathophysiological changes is increasingly being explored (Kanno et al., 2012; Maiese, 2014). mTOR is a serine/threonine kinase that functions through two protein complexes: mTORC1 and mTORC2 (Yang et al., 2013). Inhibition of the mTORC1 signaling pathway by rapamycin can enhance autophagy and promote neural repair and reconstruction after SCI (Goldshmit et al., 2015; Vargova et al., 2021). Compared with that of mTORC1, the functional relevance of mTORC2 is unclear in SCI. Rictor is a core component of the mTORC2 complex and is insensitive to rapamycin (Sarbassov et al., 2004; Wong et al., 2025). Previously, we demonstrated that the lentivirus-induced overexpression of Rictor in rats can effectively reduce the acute inflammatory response and cell death after SCI, suggesting that Rictor is a potentially novel target for the treatment of SCI (Chen et al., 2020). It was recently reported that Rictor exerts varying degrees of metabolic regulation by stabilizing mitochondria in human embryonic stem cells and human mesenchymal stem cells but not in human NSCs (Chu et al., 2022). However, Rictor deficiency adversely affects both self-renewal and differentiation abilities in all three human stem cell types (Chu et al., 2022). To investigate the role of Rictor/mTORC2 in eNSCs after SCI, we first generated mice with the eNSC-specific deletion of the Rictor gene using the Cre-loxP system. We observed no clear pathological phenotypic changes in the spinal cord using hematoxylin and eosin and Nissl staining, indicating that Rictor/mTORC2 is not essential for eNSC survival or spinal cord development under physiological conditions in mice. However, the *Nes-Cre;Rictor*^*flox/flox*^ mice showed markedly slower functional recovery and greater lesions after SCI, and more M1-polarized macrophages and fewer M2-polarized macrophages were detected in the lesion, indicating a higher level of inflammation. Together, these results suggest that Rictor/mTORC2 is a pro-survival factor during SCI repair.

As a marker of astrocytes, GFAP is often used to evaluate glial scars after SCI. Our data demonstrated that *Nes-Cre;Rictor*^*flox/flox*^ mice exhibited larger glial scars in the lesion than WT mice after SCI. We speculate that Rictor deficiency in eNSCs may hinder early glial scar formation with unrestricted inflammation, causing more widespread damage and inducing more activated astrocytes to form larger glial scars. Glial scarring is a major obstacle to axonal regeneration and neural network connectivity in the later stages of repair (Adams and Gallo, 2018). Moreover, our results revealed that fewer regenerative axons emerged in the lesions of the *Nes-Cre;Rictor*^*flox/flox*^ mice, which may have been related to the increased size of the glial scars.

Cell death is a key mechanism in secondary injury after SCI (Quadri et al., 2020). A previous study has proposed that apoptosis and necrosis are the major modes of cell death that occur during SCI (Beattie et al., 2002). Recently, however, multiple programmed cell death pathways have been identified to be involved in the process of SCI. To date, necroptosis, pyroptosis, and autophagy have all been extensively studied in the context of SCI (Shi et al., 2021). Our previous study revealed that eNSCs recruited to the lesion undergo necroptosis after SCI, and that inhibiting necroptosis of eNSCs can promote repair after SCI (Tong et al., 2023). Necroptosis is a pathway of programmed cell necrosis that is regulated by the RIPK1/RIPK3/MLKL signaling cascade, which induces a proinflammatory state (Galluzzi et al., 2017). Specifically, RIPK1 interacts with RIPK3 through its RIP homotypic interaction motif and then spontaneously phosphorylates during necroptosis induction; this leads to the phosphorylation of downstream MLKL and the formation of oligomers. These oligomers translocate to the plasma membrane and disrupt the plasma membrane phospholipid bimolecular structure to form pores (Degterev et al., 2008; Wang et al., 2014). On the pathological level, necroptotic cells swell, leak, rupture, and release damage-associated molecular patterns to promote inflammatory reactions (Degterev et al., 2005). In the present study, the ablation of Rictor after SCI sensitized eNSCs to necroptosis rather than apoptosis or pyroptosis.

To further elucidate the underlying mechanism for the pro-survival effect of Rictor/mTORC2 in eNSCs after SCI, we knocked down Rictor expression in NSCs using lentivirus-shRictor *in vitro*. The simple ablation of Rictor did not lead to RIPK1/p-MLKL-mediated necroptosis in NSCs, suggesting that Rictor does not directly regulate RIPK1 expression. Next, we subjected NSCs to combined treatment with TNF-α and LPS to simulate the inflammatory environment of SCI. Similar to the *in vivo* experiments, the necroptosis of NSCs with Rictor knockdown was significantly increased after 24 hours of co-treatment with TNF-α and LPS, suggesting that the loss of Rictor in NSCs in an inflammatory environment promotes cell necroptosis.

Under stressful conditions, activated autophagy‒lysosomes are an adaptive response to degrade self-cytoplasmic proteins and damaged organelles; this is important for maintaining cellular homeostasis and promoting cell survival (Luzio et al., 2007). Accumulation of the necroptosis-regulating kinases RIPK1 and RIPK3 is closely linked to autophagy‒lysosomes (Alu et al., 2020). Lysosomal damage after SCI causes the accumulation of RIPK1 and RIPK3 and potentiates necroptosis (Liu et al., 2018). Autophagy-initiating kinase ULK1 inhibits TNF-induced necroptosis by phosphorylating RIPK1 within its intermediate domain at Ser357 (Wu et al., 2020). The LC3-interacting region domain mediates the direct interaction of RIPK1 and RIPK3. RIPK1 and RIPK3 are cleared through the autophagy pathway to inhibit necroptosis when autophagy is activated (Huang et al., 2021). The potentiation of necroptosis followed by the inhibition of autophagy‒lysosomes is reportedly detrimental to cell survival and exacerbates damage in neurodegenerative diseases (Nixon, 2013; Menzies et al., 2017; Liu et al., 2018). In the present study, Rictor deficiency under inflammatory conditions impaired lysosomal function, which resulted in the disruption of autophagic flux and the accumulation of RIPK1. Collectively, these findings suggest that the mechanism underlying Rictor deficiency-induced necroptotic sensitization involves lysosomal damage.

mTORC2/Rictor plays an essential role in the autophagy–lysosome pathway. L-type amino acid transporter 1 directly interacts with Rictor and recruits the mTORC2 complex to the lysosome, thus facilitating cell migration (Tae et al., 2023). Phosphorylation of the key downstream substrates of mTORC2—AKT and serum glucocorticoid-inducible kinase 1—can further promote phosphorylation of the forkhead box O transcription factors, thus influencing their cellular localization and transcriptional activity, which suppresses the expression of autophagy-related genes (Ballesteros-Álvarez and Andersen, 2021). In particular, AKT phosphorylation at Ser473 has been revealed to be involved in the chaperone-mediated autophagy process mediated by LAMP2A. In Parkinson’s disease, α-synuclein degradation is regulated through AKT phosphorylation and the interaction between mTORC2 and PH domain leucine-rich repeat-containing protein phosphatase 1, thereby influencing lysosomal function and improving the chaperone-mediated autophagy process (Arias et al., 2015). A recent study has highlighted the functional coupling between lysosomal cation release and Ca^2+^ influx, which is a crucial mechanism for maintaining dopamine neuron function (Gregori et al., 2025). Rictor is mainly located in the endoplasmic reticulum, and Rictor deficiency disrupts endoplasmic reticulum Ca^2+^ homeostasis and leads to excessive cytosolic Ca^2+^ accumulation (Boulbés et al., 2011; Betz et al., 2013). Our results suggest that Rictor deficiency significantly increases the concentration of cytosolic Ca^2+^ in NSCs under inflammatory conditions, which may be related to lysosomal damage. A TNF-induced increase in cytosolic Ca^2+^ reportedly collapses the lysosomal membrane potential and induces lysosomal enlargement, leading to low degradation (Ono et al., 2003; Li et al., 2008). Notably, the decrease in lysosomal function in Rictor-deficient NSCs under inflammatory conditions in our study was rescued by treatment with the AKT phosphorylation agonist SC79 and the cell-permeable Ca^2+^ chelator BAPTA-AM, further supporting the involvement of AKT signaling and Ca^2+^ regulation in lysosomal function. Future research should focus on identifying the precise molecular interactions between Rictor/mTORC2 and key autophagy-related proteins, as well as their effects on lysosomal membrane integrity and function. Additionally, investigating the role of Ca^2+^ signaling in Rictor-deficient cells and its effects on lysosomal function may provide valuable insights into the regulation of lysosomal activity under inflammatory conditions.

This study has several limitations. Our study primarily focuses on validation and exploration through Rictor knockout. In future research, we aim to construct conditional overexpression or constitutive activation models of Rictor or other mTORC2 components specifically in eNSCs for further reverse validation. Additionally, while we confirmed *in vitro* that the cell-permeable Ca^2+^ chelator BAPTA-AM and the AKT phosphorylation agonist SC79 can restore lysosomal function and reduce necroptosis, their dosage, safety, and therapeutic efficacy still require validation at the animal level, which could ultimately lead to the development of targeted drugs or delivery materials.

Taken together, these results suggest that Rictor/mTORC2 regulation plays a critical role in the survival of eNSCs in SCI repair. In the present study, Rictor deficiency impaired lysosomal function by inhibiting AKT phosphorylation, thereby increasing RIPK1-dependent necroptotic sensitization. Importantly, this Rictor deficiency-mediated lysosomal impairment in NSCs may have been caused by the excessive release of cytosolic Ca^2+^. These findings suggest that regulating Rictor/mTORC2 in SCI repair may help to reduce eNSC necroptosis and promote eNSC survival. Notably, our findings regarding Rictor/mTORC2 in lysosomal protection and necroptosis suppression require further validation via *in vivo* overexpression. Future studies will explore the therapeutic potential of targeting this pathway for SCI repair.

## Additional files:

***Additional Figure 1:*** *eNSC-specific deletion of Rictor amplifies neuronal loss post-SCI.*

Additional Figure 1 eNSC-specific deletion of Rictor amplifies neuronal loss post-SCI.
(A) Immunofluorescence staining showing the neurons that were labeled by NeuN (red, Alexa Fluor 594) in the spinal cord. Notably, in the sham group, there was no significant difference in neuron number in the spinal cord between WT mice and Nes-Cre;Rictor^flox/flox^ mice; however, after SCI, the number of neurons in the lesion decreased significantly. In addition, compared with the findings in the WT SCI group, there were fewer labeled neurons in the Nes-Cre;Rictor^flox/flox^ group. Scale bars: 500 μm, 50 μm. (B) Quantification of NeuN^+^ cells in the spinal cord. All data are presented as the mean ± SD (*n* = 3). ^*^P < 0.05 (one-way analysis of variance followed by the Bonferroni correction for multiple comparisons). DAPI: 4',6-Diamidino-2-phenylindole; eNSC: endogenous neural stem cell; SCI: spinal cord injury; WT: wild-type.

***Additional Figure 2:*** *Effect of eNSC-specific Rictor deletion on other cell death pathways.*

Additional Figure 2 Effects of eNSC-specific Rictor deletion on other cell death pathways.
(A) Immunofluorescence staining showing nestin^+^ (red, Alexa Fluor 594) and TUNEL^+^ (green, Alexa Fluor 488) cells in lesions of the spinal cord at 14 days post-SCI. Under SCI conditions, Rictor deletion did not significantly affect nestin/TUNEL cells. Scale bars: 500 μm, 50 μm. (B) Quantitative determination of nestin ^+^TUNEL^+^ cells in the lesion among WT and Nes-Cre;Rictor^flox/flox^ mice. (C) Immunofluorescence staining showing nestin^+^ (green, Alexa Fluor 488) and GSDMD^+^ (red, Alexa Fluor 594) cells in spinal cord lesions at 14 days post-SCI. Under SCI conditions, Rictor deletion did not significantly affect nestin/GSDMD cells. Scale bars: 500 μm, 50 μm (enlarged images). (D) Quantitative determination of nestin^+^GSDMD^+^ cells in the lesion among WT and *Nes-Cre*;Rictor^flox/flox^ mice. All data are presented as the mean ± SD (*n* = 3), and were analyzed using two-tailed unpaired Student’s *t*-test. DAPI: 4',6-Diamidino-2-phenylindole; eNSC: endogenous neural stem cell; GSDMD: gasdermin D; ns: not significant; SCI: spinal cord injury; TUNEL: terminal deoxynucleotidyl transferase dUTP nick end; WT: wild-type.

***Additional Figure 3:*** *Identification and characterization of NSCs.*

Additional Figure 3 Identification and characterization of NSCs.
(A) Double immunofluorescence staining of nestin (red, Alexa Fluor 594) and SOX2 (green, Alexa Fluor 488) in NSCs. Scale bar: 25 μm. (B) NSCs were extracted from the spinal cords of newborn mice and observed under a light microscope. The NSCs grew in suspension and proliferated into spheres (10× magnification). (C) Double immunofluorescence staining of nestin (red, Alexa Fluor 594) and SOX2 (green, Alexa Fluor 488) in shC-NSCs and shRictor-NSCs. Rictor knockdown did not significantly alter the expression of nestin or SOX2. Scale bar: 50 μm. DAPI: 4',6-Diamidino-2-phenylindole; NSC: neural stem cell; SOX2: SRY-box transcription factor 2.

***Additional Figure 4:*** *Knockdown of Rictor impairs the lysosomal function of NSCs.*

Additional Figure 4 Knockdown of Rictor impairs the lysosomal function of NSCs.
Immunofluorescence staining showing the expression of RIPK1 (red, Alexa Fluor 594) and LAMP2B (green, Alexa Fluor 488) in shC-NSCs and shRictor-NSCs after 24 hours of TL treatment. The expression of RIPK1 was significantly increased in shRictor-NSCs after 24 hours of TL treatment, whereas LAMP2B expression was significantly decreased. Scale bar: 20 μm. DMSO: Dimethyl sulfoxide; LAMP2B: lysosomal-associated membrane protein 2B; LPS: lipopolysaccharide; NSC: neural stem cell; RIPK1: receptor interacting protein kinase 1; TL: TNF-α + LPS; TNF-α: tumor necrosis factor-alpha.

***Additional Figure 5:*** *Autophagy-lysosome and mTORC2 signaling pathways in response to Rictor knockdown.*

Additional Figure 5 Autophagy-lysosome and mTORC2 signaling pathways in response to Rictor knockdown.
(A) Expression of P62, LC3A/B, RIPK1, and p-MLKL in shC-NSCs and shRictor-NSCs after 24 hours of TL and TL + CQ treatment. GAPDH was used as a loading control. (B) Expression of AKT and p-AKT in shC-NSCs and shRictor-NSCs after 24 hours of TL and TL + SC79 treatment. GAPDH was used as a loading control. Akt: Protein kinase B; CQ: chloroquine; GAPDH: glyceraldehyde-3-phosphate-dehydrogenase; LC3A/B: microtubule-associated protein 1 light chain 3 A/B; LPS: lipopolysaccharide; mTORC2: mammalian target of rapamycin complex 2; NSC: neural stem cell; p-Akt: phospho-protein kinase B; pMLKL: phospho-mixed lineage kinase domain-like protein; RIPK1: receptor interacting protein kinase 1; TL: TNF-α + LPS; TNF-α: tumor necrosis factor-alpha.

## Data Availability

*All data generated or analyzed in this study are included in this published article and its Additional files.*

## References

[R1] Adams KL, Gallo V (2018). The diversity and disparity of the glial scar. Nat Neurosci.

[R2] Alfaro-Cervello C, Soriano-Navarro M, Mirzadeh Z, Alvarez-Buylla A, Garcia-Verdugo JM (2012). Biciliated ependymal cell proliferation contributes to spinal cord growth. J Comp Neurol.

[R3] Allen AR (1911). Surgery of experimental lesion of spinal cord equivalent to crush injury of fracture dislocation of spinal column: a preliminary report. J Am Med Assoc.

[R4] Alu A, Han X, Ma X, Wu M, Wei Y, Wei X (2020). The role of lysosome in regulated necrosis. Acta Pharm Sin B.

[R5] An P, Xu W, Luo J, Luo Y (2021). Expanding TOR complex 2 signaling: emerging regulators and new connections. Front Cell Dev Biol.

[R6] Arias E, Koga H, Diaz A, Mocholi E, Patel B, Cuervo AM (2015). Lysosomal mTORC2/PHLPP1/Akt regulate chaperone-mediated autophagy. Mol Cell.

[R7] Ballesteros-Álvarez J, Andersen JK (2021). mTORC2: The other mTOR in autophagy regulation. Aging Cell.

[R8] Basso DM, Beattie MS, Bresnahan JC (1995). A sensitive and reliable locomotor rating scale for open field testing in rats. J Neurotrauma.

[R9] Beattie MS, Hermann GE, Rogers RC, Bresnahan JC (2002). Cell death in models of spinal cord injury. Prog Brain Res.

[R10] Bertheloot D, Latz E, Franklin BS (2021). Necroptosis, pyroptosis and apoptosis: an intricate game of cell death. Cell Mol Immunol.

[R11] Betz C, Stracka D, Prescianotto-Baschong C, Frieden M, Demaurex N, Hall MN (2013). Feature article: mTOR complex 2-Akt signaling at mitochondria-associated endoplasmic reticulum membranes (MAM) regulates mitochondrial physiology. Proc Natl Acad Sci U S A.

[R12] Boulbés DR, Shaiken T, Sarbassov dos D (2011). Endoplasmic reticulum is a main localization site of mTORC2. Biochem Biophys Res Commun.

[R13] Chen CH, Shaikenov T, Peterson TR, Aimbetov R, Bissenbaev AK, Lee SW, Wu J, Lin HK, Sarbassov dos D (2011). ER stress inhibits mTORC2 and Akt signaling through GSK-3β-mediated phosphorylation of rictor. Sci Signal.

[R14] Chen G, Li X, Zhu H, Wu H, He D, Shi L, Wei F, Liu X, Chen N, Liu S (2022). Transplanting neurofibromatosis-1 gene knockout neural stem cells improve functional recovery in rats with spinal cord injury by enhancing the mTORC2 pathway. Exp Mol Med.

[R15] Chen N, Zhou P, Liu X, Li J, Wan Y, Liu S, Wei F (2020). Overexpression of Rictor in the injured spinal cord promotes functional recovery in a rat model of spinal cord injury. FASEB J.

[R16] Chu Q, Liu F, He Y, Jiang X, Cai Y, Wu Z, Yan K, Geng L, Zhang Y, Feng H, Zhou K, Wang S, Zhang W, Liu GH, Ma S, Qu J, Song M (2022). mTORC2/RICTOR exerts differential levels of metabolic control in human embryonic, mesenchymal and neural stem cells. Protein Cell.

[R17] Clifford T, Finkel Z, Rodriguez B, Joseph A, Cai L (2023). Current advancements in spinal cord injury research-glial scar formation and neural regeneration. Cells.

[R18] Cofano F, Boido M, Monticelli M, Zenga F, Ducati A, Vercelli A, Garbossa D (2019). Mesenchymal stem cells for spinal cord injury: current options, limitations, and future of cell therapy. Int J Mol Sci.

[R19] Darian-Smith C (2009). Synaptic plasticity, neurogenesis, and functional recovery after spinal cord injury. Neuroscientist.

[R20] Degterev A, Huang Z, Boyce M, Li Y, Jagtap P, Mizushima N, Cuny GD, Mitchison TJ, Moskowitz MA, Yuan J (2005). Chemical inhibitor of nonapoptotic cell death with therapeutic potential for ischemic brain injury. Nat Chem Biol.

[R21] Degterev A, Hitomi J, Germscheid M, Ch’en IL, Korkina O, Teng X, Abbott D, Cuny GD, Yuan C, Wagner G, Hedrick SM, Gerber SA, Lugovskoy A, Yuan J (2008). Identification of RIP1 kinase as a specific cellular target of necrostatins. Nat Chem Biol.

[R22] Dhaliwal NK, Muffat J, Li Y (2026). mTORC1 and mTORC2 synergy in human neural development, disease, and regeneration. Neural Regen Res.

[R23] Eckert MJ, Martin MJ (2017). Trauma: Spinal cord injury. Surg Clin North Am.

[R24] Fitch MT, Silver J (2008). CNS injury, glial scars, and inflammation: Inhibitory extracellular matrices and regeneration failure. Exp Neurol.

[R25] Galluzzi L, Kepp O, Chan FK, Kroemer G (2017). Necroptosis: Mechanisms and relevance to disease. Annu Rev Pathol.

[R26] Gampierakis IA, Koutmani Y, Semitekolou M, Morianos I, Polissidis A, Katsouda A, Charalampopoulos I, Xanthou G, Gravanis A, Karalis KP (2021). Hippocampal neural stem cells and microglia response to experimental inflammatory bowel disease (IBD). Mol Psychiatry.

[R27] Gazdic M, Volarevic V, Harrell CR, Fellabaum C, Jovicic N, Arsenijevic N, Stojkovic M (2018). Stem cells therapy for spinal cord injury. Int J Mol Sci.

[R28] Goldshmit Y, Kanner S, Zacs M, Frisca F, Pinto AR, Currie PD, Pinkas-Kramarski R (2015). Rapamycin increases neuronal survival, reduces inflammation and astrocyte proliferation after spinal cord injury. Mol Cell Neurosci.

[R29] Goul C, Peruzzo R, Zoncu R (2023). The molecular basis of nutrient sensing and signalling by mTORC1 in metabolism regulation and disease. Nat Rev Mol Cell Biol.

[R30] Grégoire CA, Goldenstein BL, Floriddia EM, Barnabé-Heider F, Fernandes KJ (2015). Endogenous neural stem cell responses to stroke and spinal cord injury. Glia.

[R31] Gregori M, Pereira GJS, Allen R, West N, Chau KY, Cai X, Bostock MP, Bolsover SR, Keller M, Lee CY, Lei SH, Harvey K, Bracher F, Grimm C, Hasan G, Gegg ME, Schapira AHV, Sweeney ST, Patel S (2025). Lysosomal TPC2 channels disrupt Ca2+ entry and dopaminergic function in models of LRRK2-Parkinson’s disease. J Cell Biol.

[R32] Hosseini SM, Borys B, Karimi-Abdolrezaee S (2024). Neural stem cell therapies for spinal cord injury repair: an update on recent preclinical and clinical advances. Brain.

[R33] Huang Y, Feng Y, Cui L, Yang L, Zhang Q, Zhang J, Jiang X, Zhang X, Lv Y, Jia JZ, Zhang DX, Huang YS (2021). Autophagy-related LC3 accumulation interacted directly with LIR containing RIPK1 and RIPK3, stimulating necroptosis in hypoxic cardiomyocytes. Front Cell Dev Biol.

[R34] Jo H, Mondal S, Tan D, Nagata E, Takizawa S, Sharma AK, Hou Q, Shanmugasundaram K, Prasad A, Tung JK, Tejeda AO, Man H, Rigby AC, Luo HR (2012). Small molecule-induced cytosolic activation of protein kinase Akt rescues ischemia-elicited neuronal death. Proc Natl Acad Sci U S A.

[R35] Kanno H, Ozawa H, Sekiguchi A, Yamaya S, Tateda S, Yahata K, Itoi E (2012). The role of mTOR signaling pathway in spinal cord injury. Cell Cycle.

[R36] Li C, Luo Y, Li S (2024). The roles of neural stem cells in myelin regeneration and repair therapy after spinal cord injury. Stem Cell Res Ther.

[R37] Li C, Liu Q, Li N, Chen W, Wang L, Wang Y, Yu Y, Cao X (2008). EAPF/Phafin-2, a novel endoplasmic reticulum-associated protein, facilitates TNF-alpha-triggered cellular apoptosis through endoplasmic reticulum-mitochondrial apoptotic pathway. J Mol Med (Berl).

[R38] Liu Q, Peng S, Tang Q, Li C, Chen J, Pang P, Liu W, Zhou X, Cai X, Lin H, Xue W, Ji X, Ji Z (2024). A N-Cadherin nano-antagonist hydrogel enhances recovery from spinal cord injury by impeding glial scarring. Adv Funct Mater.

[R39] Liu S, Li Y, Choi HMC, Sarkar C, Koh EY, Wu J, Lipinski MM (2018). Lysosomal damage after spinal cord injury causes accumulation of RIPK1 and RIPK3 proteins and potentiation of necroptosis. Cell Death Dis.

[R40] Lloyd-Evans E, Waller-Evans H (2020). Lysosomal Ca(2+) homeostasis and signaling in health and disease. Cold Spring Harb Perspect Biol.

[R41] Luzio JP, Pryor PR, Bright NA (2007). Lysosomes: fusion and function. Nat Rev Mol Cell Biol.

[R42] Maiese K (2014). Driving neural regeneration through the mammalian target of rapamycin. Neural Regen Res.

[R43] Mauthe M, Orhon I, Rocchi C, Zhou X, Luhr M, Hijlkema KJ, Coppes RP, Engedal N, Mari M, Reggiori F (2018). Chloroquine inhibits autophagic flux by decreasing autophagosome-lysosome fusion. Autophagy.

[R44] McDonald JW, Sadowsky C (2002). Spinal-cord injury. Lancet.

[R45] Menzies FM (2017). Autophagy and neurodegeneration: pathogenic mechanisms and therapeutic opportunities. Neuron.

[R46] Mothe AJ, Tator CH (2005). Proliferation, migration, and differentiation of endogenous ependymal region stem/progenitor cells following minimal spinal cord injury in the adult rat. Neuroscience.

[R47] Nixon RA (2013). The role of autophagy in neurodegenerative disease. Nat Med.

[R48] Ono K, Kim SO, Han J (2003). Susceptibility of lysosomes to rupture is a determinant for plasma membrane disruption in tumor necrosis factor alpha-induced cell death. Mol Cell Biol.

[R49] Percie du Sert N (2020). The ARRIVE guidelines 2.0: Updated guidelines for reporting animal research. PLoS Biol.

[R50] Pfenninger CV, Steinhoff C, Hertwig F, Nuber UA (2011). Prospectively isolated CD133/CD24-positive ependymal cells from the adult spinal cord and lateral ventricle wall differ in their long-term in vitro self-renewal and in vivo gene expression. Glia.

[R51] Qiao L, Hu J, Qiu X, Wang C, Peng J, Zhang C, Zhang M, Lu H, Chen W (2023). LAMP2A, LAMP2B and LAMP2C: similar structures, divergent roles. Autophagy.

[R52] Quadri SA, Farooqui M, Ikram A, Zafar A, Khan MA, Suriya SS, Claus CF, Fiani B, Rahman M, Ramachandran A, Armstrong IIT, Taqi MA, Mortazavi MM (2020). Recent update on basic mechanisms of spinal cord injury. Neurosurg Rev.

[R53] Rodriguez-Vargas JM, Martin-Hernandez K, Wang W, Kunath N, Suganthan R, Amé JC, Oliver FJ, Ye J, Bjørås M, Dantzer F (2020). Parp3 promotes astrocytic differentiation through a tight regulation of Nox4-induced ROS and mTorc2 activation. Cell Death Dis.

[R54] Rong Y, Liu W, Wang J, Fan J, Luo Y, Li L, Kong F, Chen J, Tang P, Cai W (2019). Neural stem cell-derived small extracellular vesicles attenuate apoptosis and neuroinflammation after traumatic spinal cord injury by activating autophagy. Cell Death Dis.

[R55] Sabelström H, Stenudd M, Frisén J (2014). Neural stem cells in the adult spinal cord. Exp Neurol.

[R56] Sabelström H, Stenudd M, Réu P, Dias DO, Elfineh M, Zdunek S, Damberg P, Göritz C, Frisén J (2013). Resident neural stem cells restrict tissue damage and neuronal loss after spinal cord injury in mice. Science.

[R57] Sánchez-Martín P, Saito T, Komatsu M (2019). p62/SQSTM1: ‘Jack of all trades’ in health and cancer. FEBS J.

[R58] Sarbassov DD, Ali SM, Kim DH, Guertin DA, Latek RR, Erdjument-Bromage H, Tempst P, Sabatini DM (2004). Rictor, a novel binding partner of mTOR, defines a rapamycin-insensitive and raptor-independent pathway that regulates the cytoskeleton. Curr Biol.

[R59] Shao A, Tu S, Lu J, Zhang J (2019). Crosstalk between stem cell and spinal cord injury: pathophysiology and treatment strategies. Stem Cell Res Ther.

[R60] Shi Z, Yuan S, Shi L, Li J, Ning G, Kong X, Feng S (2021). Programmed cell death in spinal cord injury pathogenesis and therapy. Cell Prolif.

[R61] Tae K, Kim SJ, Cho SW, Lee H, Cha HS, Choi CY (2023). L-type amino acid transporter 1 (LAT1) promotes PMA-induced cell migration through mTORC2 activation at the lysosome. Cells.

[R62] Tang X, Deng P, Li L, He Y, Wang J, Hao D, Yang H (2024). Advances in genetically modified neural stem cell therapy for central nervous system injury and neurological diseases. Stem Cell Res Ther.

[R63] Tong K, Li S, Chen G, Ma C, Liu X, Liu S, Chen N (2023). Inhibition of neural stem cell necroptosis mediated by RIPK1/MLKL promotes functional recovery after SCI. Mol Neurobiol.

[R64] Tran AP, Warren PM, Silver J (2022). New insights into glial scar formation after spinal cord injury. Cell Tissue Res.

[R65] Udayar V, Chen Y, Sidransky E, Jagasia R (2022). Lysosomal dysfunction in neurodegeneration: emerging concepts and methods. Trends Neurosci.

[R66] Vargova I, Machova Urdzikova L, Karova K, Smejkalova B, Sursal T, Cimermanova V, Turnovcova K, Gandhi CD, Jhanwar-Uniyal M, Jendelova P (2021). Involvement of mTOR pathways in recovery from spinal cord injury by modulation of autophagy and immune response. Biomedicines.

[R67] Venkatesh K, Ghosh SK, Mullick M, Manivasagam G, Sen D (2019). Spinal cord injury: pathophysiology, treatment strategies, associated challenges, and future implications. Cell Tissue Res.

[R68] Wang H, Sun L, Su L, Rizo J, Liu L, Wang LF, Wang FS, Wang X (2014). Mixed lineage kinase domain-like protein MLKL causes necrotic membrane disruption upon phosphorylation by RIP3. Mol Cell.

[R69] Weiss S, Dunne C, Hewson J, Wohl C, Wheatley M, Peterson AC, Reynolds BA (1996). Multipotent CNS stem cells are present in the adult mammalian spinal cord and ventricular neuroaxis. J Neurosci.

[R70] Wie MB, Koh JY, Won MH, Lee JC, Shin TK, Moon CJ, Ha HJ, Park SM, Kim HC (2001). BAPTA/AM, an intracellular calcium chelator, induces delayed necrosis by lipoxygenase-mediated free radicals in mouse cortical cultures. Prog Neuropsychopharmacol Biol Psychiatry.

[R71] Wong C, Rodriguez-Hernandez LD, Lister KC, Gu N, Cai W, Hooshmandi M, Fan J, Brown N, Nguyen V, Ribeiro-da-Silva A, Bonin RP, Khoutorsky A (2025). Targeting spinal mechanistic target of rapamycin complex 2 alleviates inflammatory and neuropathic pain. Brain.

[R72] Wu H, Tong K, Liu X, Li J, Li X, Gao M, Tian W, Chen D, Zhou Z, Liu S (2022). A comparison between two laminectomy procedures in mouse spinal cord injury on Allen’s animal model. J Neurosci Methods.

[R73] Wu W, Wang X, Berleth N, Deitersen J, Wallot-Hieke N, Böhler P, Schlütermann D, Stuhldreier F, Cox J, Schmitz K, Seggewiß S, Peter C, Kasof G, Stefanski A, Stühler K, Tschapek A, Gödecke A, Stork B (2020). The autophagy-initiating kinase ULK1 controls RIPK1-mediated cell death. Cell Rep.

[R74] Yang H, Rudge DG, Koos JD, Vaidialingam B, Yang HJ, Pavletich NP (2013). mTOR kinase structure, mechanism and regulation. Nature.

